# Supra‐Fluorophores: Ultrabright Fluorescent Supramolecular Assemblies Derived from Conventional Fluorophores in Water

**DOI:** 10.1002/adma.202401346

**Published:** 2024-04-02

**Authors:** Yuqing Lei, Yuqian Wang, Sophie K. Hill, Zihe Cheng, Qiao Song, Sébastien Perrier

**Affiliations:** ^1^ Shenzhen Grubbs Institute Southern University of Science and Technology Shenzhen 518055 China; ^2^ Department of Chemistry University of Warwick Coventry CV4 7AL UK; ^3^ Warwick Medical School University of Warwick Coventry CV4 7AL UK; ^4^ Faculty of Pharmacy and Pharmaceutical Sciences Monash University Parkville VIC 3052 Australia

**Keywords:** aggregation‐caused quenching, conventional fluorophore, cyclic peptide, fluorescence quenching by water, fluorescent nanoparticle, supramolecular chemistry

## Abstract

Fluorescent organic nanoparticles (NPs) with exceptional brightness hold significant promise for demanding fluorescence bioimaging applications. Although considerable efforts are invested in developing novel organic dyes with enhanced performance, augmenting the brightness of conventional fluorophores is still one of the biggest challenges to overcome. This study presents a supramolecular strategy for constructing ultrabright fluorescent nanoparticles in aqueous media (referred to as “Supra‐fluorophores”) derived from conventional fluorophores. To achieve this, this course has employed a cylindrical nanoparticle with a hydrophobic microdomain, assembled by a cyclic peptide‐diblock copolymer conjugate in water, as a supramolecular scaffold. The noncovalent dispersion of fluorophore moieties within the hydrophobic microdomain of the scaffold effectively mitigates the undesired aggregation‐caused quenching and fluorescence quenching by water, resulting in fluorescent NPs with high brightness. This strategy is applicable to a broad spectrum of fluorophore families, covering polyaromatic hydrocarbons, coumarins, boron‐dipyrromethenes, cyanines, xanthenes, and squaraines. The resulting fluorescent NPs demonstrate high fluorescence quantum yield (>30%) and brightness per volume (as high as 12 060 m ^−1^ cm^−1^ nm^−3^). Moreover, high‐performance NPs with emission in the NIR region are constructed, showcasing up to 20‐fold increase in both brightness and photostability. This Supra‐fluorophore strategy offers a versatile and effective method for transforming existing fluorophores into ultrabright fluorescent NPs in aqueous environments, for applications such as bioimaging.

## Graphical abstract

This work presents a supramolecular strategy for constructing ultrabright fluorescent supramolecular assemblies in aqueous media (referred to as “Supra‐fluorophores”) derived from conventional fluorophores. The noncovalent dispersion of fluorophore moieties within the hydrophobic microdomain of a cyclic peptide‐based supramolecular scaffold effectively mitigates the undesired aggregation‐caused quenching and fluorescence quenching by water, resulting in Supra‐fluorophores with high brightness.

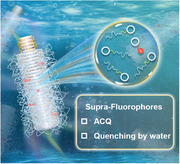

## 1 Introduction

Fluorescent materials are the key element in fluorescence bioimaging.^[^ ^1^, ^2^, ^3^ ^]^ While traditional fluorescent dyes and fluorescent proteins have been widely used for many years, they do have limitations, particularly in terms of fluorescence brightness. This limitation has driven the development of nanoscale fluorescent materials as their molar extinction coefficients can be 10–1000‐fold higher than that of molecular dyes.^[^ ^4^, ^5^, ^6^ ^]^ Among them, fluorescent organic nanoparticles (NPs) have gained attention due to their unique characteristics, including flexibility, rich surface chemistry, and biocompatibility.^[^ ^7^, ^8^, ^9^, ^10^ ^]^

To achieve exceptional brightness in fluorescent NPs, it is essential to assemble a substantial quantity of organic dyes with high fluorescence quantum yield and extinction coefficient within the limited volume of a nanoparticle.^[^ ^11^, ^12^ ^]^ However, when these common organic dyes, typically process flat aromatic structures, are present at high local concentrations within nanomaterials, they tend to form non‐emissive π‐stacked aggregates with a face‐to‐face arrangement. This phenomenon, known as aggregation‐caused quenching (ACQ), represents a major obstacle in the development of bright organic NPs.^[^ ^13^, ^14^ ^]^ In response to this challenge, one of the most popular approaches is the use of aggregation‐induced emission (AIE) dyes, proposed by Tang and co‐workers in 2001.^[^ ^15^ ^]^ The AIE concept has led to the creation of a diverse array of highly emissive nanoparticles for a wide range of applications, especially in bioimaging.^[^ ^16^, ^17^, ^18^, ^19^ ^]^ Alternatively, enhancing the brightness of conventional fluorophores by mitigating their aggregation tendencies holds equal importance. The most straightforward strategy entails the chemical modification of organic fluorophores through the introduction of bulky side groups.^[^ ^20^, ^21^, ^22^ ^]^ Yet, it is important to note that this method is highly specific and must be tailored individually for each fluorophore, rendering it unsuitable for universal application across different dye types. Supramolecular chemistry, which focuses on the fabrication of ordered structures via noncovalent interactions, offers a complementary approach to address this issue.^[^ ^23^, ^24^, ^25^, ^26^, ^27^, ^28^, ^29^, ^30^, ^31^, ^32^ ^]^ A representative strategy is provided by Klymchenko et al., who utilized bulky hydrophobic counterions to isolate ionic dyes, effectively preventing dyes from ACQ.^[^ ^33^, ^34^, ^35^, ^36^, ^37^ ^]^ The teams of Laursen and Flood presented an analogous concept called SMILES (small‐molecule ionic isolation lattices), which used a supramolecular counterion complex built of small inorganic anions with cyanostar to isolate ionic dyes.^[^ ^38^, ^39^, ^40^ ^]^ While both strategies have proven effective for dyes with positive charges, such as cyanines and rhodamines, the pursuit of a more universally applicable approach, irrespective of the dye properties, is highly appealing.

Biological imaging is predominantly conducted in aqueous environment; however, it is often overlooked that water, a ubiquitous component of biological systems, can act as a weak yet significant fluorescence quencher.^[^ ^41^, ^42^ ^]^ A systematic investigation has revealed that most fluorophores exhibit fluorescence quenching in the presence of water.^[^ ^43^ ^]^ The quenching effect is mainly attributed to Förster resonant energy transfer from the excited dye to water molecule, with a more pronounced quenching efficiency for red‐emitting fluorophores compared to blue‐emitting ones. Consequently, the enhancement of fluorescence brightness, especially for NIR fluorophores, can be achieved by effectively shielding the fluorophores from water molecules.

Self‐assembling cyclic peptides are capable of stacking into nanotubular structures through the multiple hydrogen bonding interactions.^[^ ^44^, ^45^ ^]^ When a hydrophilic polymer chain is attached to the cyclic peptide, it results in the creation of a precisely defined core‐shell cylindrical structure.^[^ ^46^, ^47^ ^]^ This precisely organized supramolecular structure has been utilized as a scaffold to molecularly align functional elements, including drugs, π‐conjugated chromophores, proteins, and even nanoparticles.^[^ ^48^, ^49^, ^50^, ^51^, ^52^, ^53^, ^54^ ^]^ For example, our group developed a cyclic peptide‐based supramolecular scaffold to inhibit the undesired cis‐trans photoisomerization and ACQ of cyanine dyes, resulting a series of ultrabright Supra‐cyanines.^[^ ^55^ ^]^ Furthermore, the attachment of an amphiphile diblock copolymer to the cyclic peptide forms a hydrophobic microdomain surrounding the cyclic peptide‐based nanotube in aqueous media.^[^ ^56^ ^]^ This led us to envision that by placing the dye molecules within this hydrophobic microdomain to shield them from water molecules, while distributing them along the cylindrical assemblies, we could sufficiently suppress undesired ACQ and fluorescence quenching by water simultaneously, thus resulting in fluorescent NPs with boosted brightness. Most importantly, this approach is believed to be universally applicable since it does not depend on the properties of the dyes.

**Scheme 1:**
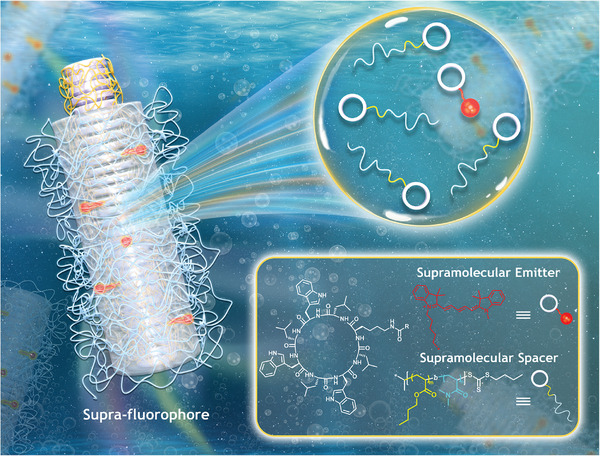
Cartoon illustration demonstrating the construction of Supra‐fluorophores by the co‐assembly of supramolecular emitter and supramolecular spacer in aqueous media.

In this work, we present a supramolecular strategy to constructing ultrabright fluorescent nanoparticles in aqueous media (referred to as “Supra‐fluorophores”) from conventional fluorophores. As illustrated in **Scheme** **1**, a cyclic peptide‐diblock copolymer conjugate is designed to act as a supramolecular spacer, capable of self‐assembling into cylindrical nanoparticles with a hydrophobic microdomain in water. The supramolecular emitter is produced by covalently attaching a fluorophore to the cyclic peptide. Supra‐fluorophores could be directly generated by co‐assembling both the Spacer and Emitter into the cylindrical assemblies. This method disperses the fluorophore moieties within the hydrophobic microdomain along the cylindrical assemblies, resulting in fluorescent NPs with high volume‐normalized brightness. Specifically, Supra‐SQ and Supra‐Cy5 achieve values of 8580 and 12 060 m ^−1^ cm^−1^ nm^−3^, respectively. Notably, our approach is applicable to a wide range of conventional fluorophores, including polyaromatic hydrocarbons, coumarins, boron‐dipyrromethenes, cyanines, xanthenes, and squaraines. Last but not least, high performance Supra‐NIR NPs are constructed, exhibiting up to 20‐fold increase in both brightness and photostability. Consequently, our Supra‐fluorophore strategy offers a versatile and effective method for converting existing fluorophores into ultrabright fluorescent supramolecular assemblies in aqueous environments.

## 2 Results and Discussion

### 2.1 Conjugates Synthesis and Self‐assembly Characterization

**Figure 1:**
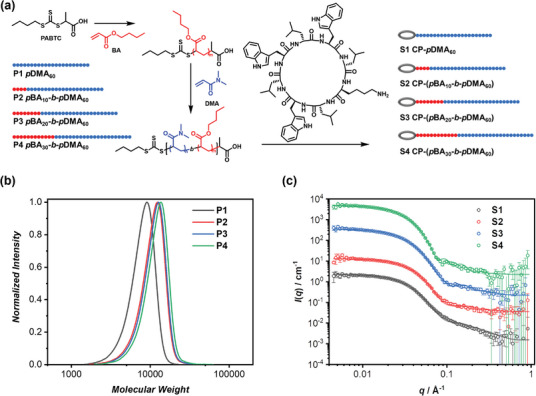
a) Synthetic procedure of supramolecular spacers **S1‐S4**; b) GPC traces of polymers **P1‐P4**; c) SANS scattering data and fitting to a core‐shell cylinder model of **S1‐S4**.

To create the supramolecular spacer for assembling into supramolecular cylindrical assemblies with a hydrophobic microdomain, an amphiphilic diblock copolymer is to be attached to the periphery of the cyclic peptide. As depicted in **Figure** **1**a, *n*‐butyl acrylate (BA) and *N*, *N*‐dimethyl acrylamide (DMA) have been selected to form the hydrophobic and hydrophilic blocks, respectively. To modulate the hydrophobic region surrounding the cyclic peptide core, diblock copolymers with varying length of the *p*BA block were synthesized using reversible addition fragmentation chain‐transfer polymerization, generating 3 diblock copolymers with narrow dispersity, denoted as *p*BA‐*b*‐*p*DMA (**P2‐P4**). Additionally, a homopolymer lacking the hydrophobic *p*BA block was synthesized for control purposes (*p*DMA, **P1**). The successful synthesis of **P1‐P4** was confirmed through gel permeation chromatography (GPC) analysis (Figure 1b, Tables S1 and S2, Supporting Information) and ^1^H NMR spectroscopy (Figure S1, Supporting Information). The spacer conjugates, denoted as **S1‐S4**, were subsequently obtained by coupling **P1‐P4** to an amino‐containing cyclic peptide via HATU coupling chemistry, which were purified through fractional precipitation (Figure S2, Table S3, Supporting Information). Concurrently, Cyanine 5 was chosen as a model dye for attachment to the same cyclic peptide, resulting CP‐Cy5 to serve as the supramolecular emitter.

The self‐assembling structures of the 4 supramolecular spacers in water were characterized using small angle neutron scattering (SANS). Figure 1c shows the reduced, corrected SANS data for **S1‐S4** in D_2_O. These data were analyzed with SasView software and fitted to a core‐shell cylindrical model, confirming the formation of supramolecular cylindrical assemblies (Table S4, Supporting Information). The fluorescent supramolecular assembly, referred to as Supra‐Cy5, was easily constructed by co‐assembling **S1‐S4** and CP‐Cy5 in aqueous solutions at specific molar ratios. The self‐assembling structure of Supra‐Cy5 was found to be similar to that of an individual spacer (Figure S3, Supporting Information).

### 2.2 Photophysical Performance of Supra‐Cy5 Built by Different Spacers

**Figure 2:**
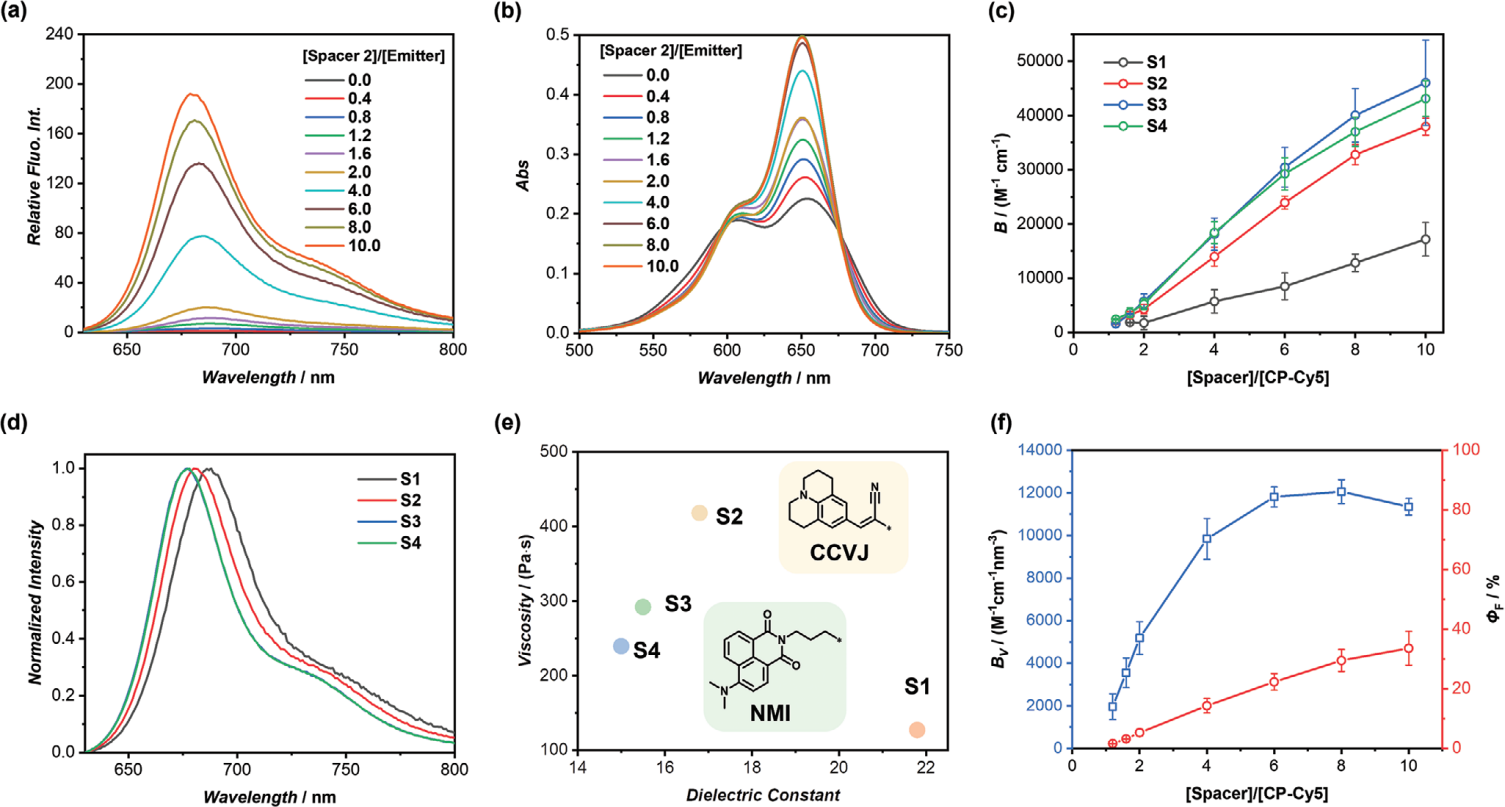
Photophysical properties of Supra‐Cy5. a) Fluorescence spectra of Supra‐Cy5 built by CP‐Cy5 and **S2** at different molar ratios; b) UV–vis absorption spectra of Supra‐Cy5 built by CP‐Cy5 and **S2** at different molar ratios; c) Evolution of brightness values of Supra‐Cy5 constructed from **S1‐S4** as a function of Spacer/Emitter molar ratios; d) Normalized fluorescence spectra of Supra‐Cy5 built by **S1‐S4** at a Spacer/Emitter molar ratio of 10/1; e) Polarity and viscosity of the hydrophobic inner region within the assemblies of **S1‐S4** probed by NMI and CCVJ; f) Brightness per volume (*B_V_*) and fluorescence quantum yields (*Φ*_F_) of Supra‐Cy5 at different **S3**/CP‐Cy5 molar ratios. The sample size is ≥3. All error bars indicate the standard deviation.

The photophysical properties of Supra‐Cy5, constructed with various supramolecular spacers, were studied. Taking **S2** as an example, the concentration of CP‐Cy5 (Emitter) was kept constant at 4 µm, while **S2** was co‐assembled with different molar equivalents, ranging from 0 to 10. As shown in **Figure** **2**a, the fluorescence of CP‐Cy5 by itself was completely quenched due to pronounced ACQ. The emission intensity exhibited a consistent increase as the Spacer/Emitter molar ratio was raised. The fluorescence enhancement reached 180‐fold at a ratio of 10/1 and plateaued at ≈250‐fold at a ratio of 40/1 (Figure S4, Supporting Information). The change in the aggregation state of Cy5 within the assemblies was evident in UV–vis spectroscopy results (Figure 2b). The increase of Spacer/Emitter molar ratio led to an increase in the absorption of Cy5 at 650 nm, suggesting that the Cy5 moieties were spatially separated with the assistance of **S2**. Furthermore, time‐resolved fluorescence spectroscopy was conducted to obtain the fluorescence lifetime of Cy5. As indicated in Figure S5, Supporting Information, free Cy5 displayed a notably short fluorescence lifetime (τ = 0.47 ns) in water, whereas Supra‐Cy5 exhibited a substantially longer lifetime (τ = 1.61 ns when Spacer/Emitter molar ratio is 10/1). Subsequently, the radiative and nonradiative rate constants (*k*_r_, *k*_nr_) were calculated (Table S5, Supporting Information). Both Supra‐Cy5 and free Cy5 demonstrated similar *k*_r_ values (1.91 × 10^8^ s^−1^ versus 2.81 × 10^8^ s^−1^), while the *k*_nr_ value of Supra‐Cy5 was 4.3 times smaller than that of free Cy5 (4.31 × 10^8^ s^−1^ versus 1.85 × 10^9^ s^−1^).

To quantitively compare the performance of Supra‐Cy5 built by the 4 Spacers, measurements and calculations of the fluorescence quantum yield (*Φ*_F_) and brightness (*B*, defined as ε*Φ_F_, where *ε* is molar extinction of Cy5) were carried out (Figure 2c, Figure S6, Table S6, Supporting Information). Both *Φ*_F_ and *B* values consistently increased with the rise in Spacer/Emitter molar ratios across all 4 Spacers, without any exceptions. Meanwhile, the presence of the hydrophobic *p*BA segment played a significant role in the performance of Supra‐Cy5. At a Spacer/Emitter molar ratio of 10/1, Supra‐Cy5 formed with **S1** exhibited a modest brightness of 17 200 m ^−1^ cm^−1^ (*Φ*_F_ = 16.7%). In contrast, Supra‐Cy5 generated using **S2** showed a significantly elevated brightness, boosting a 2.2‐fold increase (37 980 m ^−1^ cm^−1^, *Φ*_F_ = 30.7%). Increasing the length of *p*BA segment led to further increases in brightness, resulting in values of 46 060 m ^−1^ cm^−1^ (*Φ*_F_ = 33.6%) and 43 150 m ^−1^ cm^−1^ (*Φ*_F_ = 29.9%) for **S3** and **S4**, respectively. Moreover, as indicated in Figure 2d, at a Spacer/Emitter molar ratio of 10/1, the emission maximum of Supra‐Cy5 showed a gradual hypsochromic shift (up to 11 nm) with the increase in the length of the hydrophobic *p*BA block, implying a change in the microenvironment of Cy5 within assemblies (Figure S7, Supporting Information).

To gain a deeper insight into the characteristics of the hydrophobic inner region within these assemblies, we utilized two fluorophores as probes for a quantitative assessment of their polarity and viscosity. Naphthalene monoimide (NMI) is well‐known for its solvatochromic properties, with both its emission and excitation wavelengths shifting depending on the solvent's polarity.^[^ ^57^ ^]^ Moreover, a linear correlation between its emission maximum and the logarithm of the solvent's dielectric constant (log *κ*) was established (Figure S8, Supporting Information). To this end, NMI was covalently linked to the cyclic peptide to obtain CP‐NMI. Subsequently, CP‐NMI was co‐assembled with **S1‐S4** in water, effectively placing the NMI moieties within the hydrophobic inner region of the assemblies. This allowed us to determine the polarity of the inner region by measuring the emission spectrum of NMI and comparing it with the standard curve. As shown in Figure 2e, a noticeable reduction in polarity was observed when comparing **S2‐S4** in contrast to **S1**, providing further confirmation of the effect of the hydrophobic *p*BA block. Moreover, as the length of *p*BA block increased, a subtle decrease in polarity was witnessed. 9‐(2‐carboxy‐2‐cyanovinyl)julolidine (CCVJ) is recognized as a fluorescent molecular rotor, where its fluorescence is inversely proportional to its intramolecular rotation.^[^ ^58^ ^]^ The dependency of the emission intensity of CCVJ on viscosity can be accurately described by a power‐law relationship (Figure S9, Supporting Information). The viscosity of the inner region within the assembly could be determined in a similar way. As depicted in Figure 2e, **S2‐S4** exhibited higher viscosity than **S1**, with a decrease in viscosity observed as the *p*BA chain length increased. It could be concluded that an increase in the length of the *p*BA block length results in a more hydrophobic but less viscous hydrophobic region. This leads to an increase in brightness from **S1** to **S3**, followed by a decrease from **S3** to **S4**, due to the balanced contribution of both polarity and viscosity. Consequently, we chose **S3** to be the optimal supramolecular spacer to construct Supra‐fluorophores.

When utilizing fluorescent nanoparticles for bioimaging applications, the ideal materials are those that are both bright and small. In this context, it is crucial to define brightness per volume (*B*_V_), as it directly reflects the brightness of a given fluorescent nanoparticle independently of its size. The high local concentrations of dyes in Supra‐Cy5 assemblies (ranging from 410 to 2050 mm), along with high extinction coefficients and fluorescence quantum yields, will result in high *B*_V_ values. As depicted in Figure 2f, *B*_V_ exhibited an increasing trend as the Spacer/Emitter molar ratio rose from 0.4 to 8. However, when the ratio exceeded 8, a gradual decrease in *B*_V_ was observed. This decline could be attributed to the presence of excess spacers in Supra‐Cy5, causing a reduction in dye density. To our delight, the maximum *B*_V_ of Supra‐Cy5 reached 12 060 m ^−1^ cm^−1^ nm^−3^ (with a *Φ*_F_ value of 29.5%) at a Spacer/Emitter molar ratio of 8 (Table S7, Supporting Information). This value is ≈2.5 times higher than the highest *B*_v_ reported in our previous research (4710 m ^−1^ cm^−1^ nm^−3^)^[^ ^55^ ^]^ and approaches the uppermost values reported for fluorescent nanoparticles in water (6600 m ^−1^ cm^−1^ nm^−3^ by Flood, Laurson, et al.^[^ ^39^ ^]^; 19 100 m ^−1^ cm^−1^ nm^−3^ by Klymchenko, et al.^[^ ^33^ ^]^).

**Figure 3:**
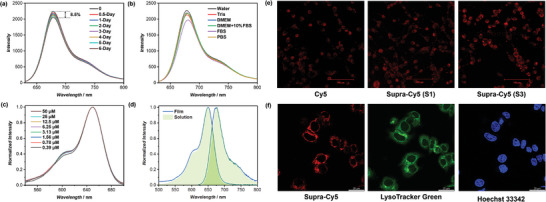
a) Fluorescence spectra of Supra‐Cy5 measured at different time intervals after preparation; b) Fluorescence spectra of Supra‐Cy5 in different aqueous media; c) Normalized UV–vis absorption spectra of Supra‐Cy5 at different concentrations; d) Normalized fluorescence excitation and emission spectra of Supra‐Cy5 in solution and the solid state; e) CLSM images of CT26 colorectal cancer cells incubated with Cy5, and Supra‐Cy5 constructed by **S1** or **S3**; f) Intracellular localization experiment showing Supra‐Cy5 (red), LysoTracker Green (green), and Hoechst 33 342 (blue) channels.

Supra‐Cy5 demonstrates remarkable stability, thanks to the strong multiple hydrogen bonding interactions between the cyclic peptides. First, we evaluated the stability of Supra‐Cy5 in water over a long period of time. As can be seen in **Figure** **3**a, only 8.5% decrease in fluorescence intensity was observed after 6 days. Second, Supra‐Cy5 exhibited excellent stability across a broad spectrum of aqueous media, including PBS, Tris, DMEM, FBS, and DMEM + 10% FBS, as evident from the consistent emission intensity shown in Figure 3b. This ensures Supra‐Cy5's suitability for a variety of bio‐related applications. Third, the concentration stability of Supra‐Cy5 was studied. Figure 3c illustrates that the concentration‐normalized UV–vis spectra remain overlapping across a wide range of Cy5 concentrations, ranging from 50 µm down to as low as 0.39 µm. This observation indicates that there is no aggregation or disassembly taking place in Supra‐Cy5 within this concentration range. Last, the photophysical properties of Supra‐Cy5 in solid states were characterized. Notably, the solid‐state Supra‐Cy5 showed nearly identical excitation and emission spectra to those obtained in water (Figure 3d), accompanied by a high *Φ*_F_ value of 33.2%. Collectively, these results underscore the impressive stability of Supra‐Cy5 in both aqueous media and the solid state.

To showcase the potential of Supra‐Cy5 for bioimaging, live CT26 colorectal cancer cells were incubated with either Supra‐Cy5 or Cy5 and imaged using confocal laser scanning microscopy (CLSM) at identical experimental conditions. As revealed in Figure 3e, Supra‐Cy5, constructed by **S3,** exhibited notably brighter fluorescence in comparison to both Cy5 or Supra‐Cy5 made from **S1**. To further elucidate the intracellular localization of Supra‐Cy5, two additional cell probes were employed: LysoTracker Green for labeling lysosomes and Hoechst 33 342 for labeling the nucleus. Figure 3f illustrates a significant overlap between the signals of Supra‐Cy5 and LysoTracker Green, with no apparent overlap between Supra‐Cy5 and Hoechst 33 342. This suggests that Supra‐Cy5 enters the cell via endocytosis without interfering with the nucleus. In summary, these experiments affirm that the remarkable brightness and stability of Supra‐Cy5 position it as an excellent candidate for fluorescent probes in bioimaging applications.

### 2.3 Expansion to 6 Categories of Organic Fluorophores

To test the effectiveness of our supramolecular strategy in a broader context, we plan to investigate 6 distinct categories of common organic fluorophores. These categories encompass polyaromatic hydrocarbons (PAHs), coumarins, boron‐dipyrromethenes (BODIPYs), cyanines, xanthenes, and squaraines, representing thousands of synthetic organic dyes. To this end, DPA, Cou343, BDP_FL, Cy3, RhB, and SQ were chosen to represent PAHs, coumarins, BODIPYs, cyanines, xanthenes, and squaraines, respectively. These fluorophores display distinct photophysical properties in water: Cou343 and BDP_FL possess near unity *Φ*_F_ values, while DPA and RhB display moderate *Φ*_F_ values; in contrast, the *Φ*_F_ values of Cy3 and SQ are exceedingly low (Table S8, Supporting Information). Furthermore, the emission spectra of these 6 fluorophores span the entire visible wavelength range (Figure S10, Supporting Information).

**Figure 4:**
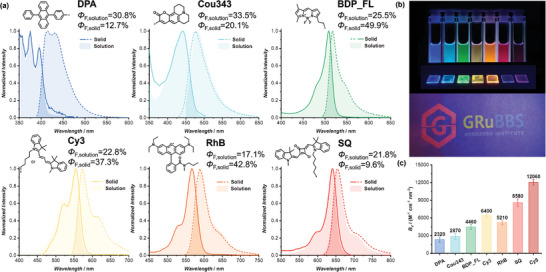
a) Normalized UV–vis absorption spectra and fluorescence emission spectra of Supra‐fluorophores in solution and solid states; b) Photographs of Supra‐fluorophores in solution and solid states (top) and printed pattern (bottom) under UV lamp; c) Summary of the maximum *B_V_* values of the Supra‐fluorophores.

Supra‐fluorophores are fabricated by co‐assembling the respective Emitters with **S3** in water using the same way as Supra‐Cy5. All of them exhibited similar increasing trends in emission intensity when the Spacer/Emitter molar ratios were increased, akin to Supra‐Cy5 (Figures S11–S16, Supporting Information). As summarized in **Figure** **4**a and Table S8, Supporting Information, both their *Φ*_F_ and *B* values reached satisfactory levels, ranging from 17.1% to 33.5%, and from 4400 to 34 690 m ^−1^ cm^−1^, respectively, at a Spacer/Emitter molar ratio of 10/1. All Supra‐fluorophores showed bright fluorescence under a UV lamp (Figure 4b). In addition, noticeable shifts were observed in both the absorption and emission spectra when comparing the Supra‐fluorophores with their small‐molecule counterparts, confirming that the fluorophores are situated within distinct microenvironments provided by the assemblies. More importantly, all Supra‐fluorophores exhibited satisfying maximum *B_V_* values, ranging from 2320 (Supra‐DPA) to 12 060 m ^−1^ cm^−1^ nm^−3^ (Supra‐Cy5) (Figure 4c). Therefore, the Supra‐fluorophore approach represents a universally applicable strategy for the construction of ultrabright nanoparticles in aqueous media which does not rely on the properties of the dyes.

The photophysical properties of the Supra‐fluorophores in solid states were then characterized. In each instance, the properties of the fluorophore were seamlessly transferred to the solid state (Figure 4a, Table S9, Supporting Information): they all exhibited not only the same fluorescence excitation and emission spectra as the Supra‐fluorophores in water, but also high fluorescence quantum yields. Therefore, we successfully obtained a series of highly emissive supra‐fluorophores in both solution and solid states, covering visible colors including blue, cyan, green, yellow, orange, red, and mahogany (Figure 4b). The Supra‐fluorophores were further utilized as fluorescent inks for fluorescence patterning. Aqueous solutions of Supra‐DPA, Supra‐BDP_FL, Supra‐Cy3, and Supra‐SQ were loaded into the ink cartridges of a commercial inkjet printer. As shown in Figure 4b, the logo and name of the Shenzhen Grubbs Institute were printed, exhibiting bright blue, green, yellow, and red fluorescence under a UV lamp. It is highly anticipated that our supramolecular strategy will yield a series of highly emissive fluorescent materials in both aqueous and solid states.

### 2.4 Supra‐NIR Dyes with High Brightness and Outstanding Photostability

NIR organic dyes play a crucial role in bioimaging due to their significance in providing imaging capabilities in the NIR region, enabling deep tissue penetration, reduced autofluorescence, and minimal photodamage.^[^ ^59^, ^60^ ^]^ Nevertheless, the majority of common NIR organic dyes encounter issues related to low fluorescence quantum yield and poor photostability.^[^ ^61^, ^62^ ^]^ Therefore, there is no doubt that developing NIR dyes with higher quantum yields and enhanced photostability will advance the field of photoimaging.

**Figure 5:**
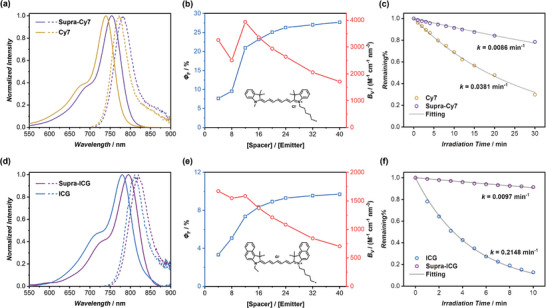
a) Normalized absorption (solid lines) and fluorescence spectra (dash lines) of Cy7 and Supra‐Cy7; b) Fluorescence quantum yields (*Φ*_F_) and brightness per volume (*B_V_*) of Supra‐Cy7 at different Spacer/Emitter molar ratios; c) Photostability of Cy7 and Supra‐Cy7 under a Xe lamp irradiation; d) Normalized absorption (solid lines) and fluorescence spectra (dash lines) of ICG and Supra‐ICG; e) Fluorescence quantum yields (*Φ*_F_) and brightness per volume (*B_V_*) of Supra‐ICG at different Spacer/Emitter molar ratios; f) Photostability of ICG and Supra‐ICG under a Xe lamp irradiation.

Recognizing the substantial impact of our supramolecular scaffold in preventing ACQ and resisting water contact, we embarked on investigating the feasibility of constructing Supra‐NIR dyes through our supramolecular approach. To this end, two of the most extensively employed NIR dyes, Cy7 and ICG, were selected. Supra‐Cy7 and Supra‐ICG were fabricated by co‐assembling CP‐Cy7 and CP‐ICG with **S3** in water, respectively. As depicted in **Figure** **5**a,d, both Supra‐Cy7 and Supra‐ICG exhibited > 10 nm bathochromic shift in both the absorption and fluorescence spectra compared to free Cy7 and ICG in water. This could be ascribed to the different microenvironment where the fluorophores are situated. The boosted emission capability was observed for both Supra‐Cy7 (Figure S17, Supporting Information) and Supra‐ICG (Figure S18, Supporting Information). The *Φ*_F_ of Cy7 and ICG in water were found to be significantly low, at only 6.4% and 0.4%. To our delight, as indicated in Figure 5b,e, when the Spacer/Emitter molar ratio was set at 20/1, the *Φ*_F_ values of Supra‐Cy7 and Supra‐ICG achieved remarkable levels of 25.1% and 8.9%, respectively. These values saw a further increase to 27.7%, and 9.7% when the molar ratio was adjusted to 40/1. As a control, Supra‐Cy7 and Supra‐ICG, constructed by **S1**, exhibited significantly lower *Φ*_F_ values (12.3% and 2.7% at 20/1; 18.6% and 5.7% at 40/1), further emphasizing the importance of the hydrophobic microenvironment. Furthermore, the maximum *B*_V_ values of Supra‐Cy7 and Supra‐ICG were also calculated, which gave 3930 and 1580 m ^−1^ cm^−1^ nm^−3^.

The photostability of Supra‐Cy7 and Supra‐ICG was then studied. For this purpose, the corresponding dye solutions were exposed to a Xe lamp, and the photobleaching of the dyes was monitored using UV–vis spectroscopy. As shown in Figure 5c, after 30 min of photoirradiation, 70% of Cy7 was photobleached, while only 22% photobleaching was observed in case of Supra‐Cy7. To quantitively access photostability, we calculated the rate constants of the photodegradation reaction. The rate constant for Supra‐Cy7 was 0.0086 min^−1^, 4.4 times smaller than that of Cy7 (0.0381 min^−1^). The disparity was even more pronounced in case of ICG. After 10 min of irradiation, ICG exhibited an 87% of photobleaching, while Supra‐ICG only showed a 9% decrease in absorption under the same conditions. An analysis of the data reveals a remarkable 22‐fold increase in the photostability of Supra‐ICG compared to ICG (Figure 5f). One of the primary mechanisms leading to photobleaching involves the dye transitioning into a triplet‐excited state through intersystem crossing and subsequently reacting with ground‐state oxygen (or other species) to generate oxygen radicals (or other highly reactive species), which in turn react with the dye, causing permanent damage. Hence, the following two factors are believed to contribute to the enhanced stability. First, the ISC process to the triplet‐excited state is greatly inhibited. Second, the hydrophobic microenvironment created by the scaffold prevents direct contact between the dye and ground‐state oxygen (or other species). Therefore, our supramolecular strategy provides an effective approach to fabricating NIR fluorescent NPs with high brightness and outstanding photostability.

## 3 Conclusion

We report a supramolecular approach for fabricating ultrabright fluorescent NPs from conventional fluorophores. By employing a unique cylindrical nanoparticle with a hydrophobic microdomain as the supramolecular scaffold, we simultaneously prevent the undesired ACQ and fluorescence quenching by water of fluorophores. This enables the facile assembly of a series of ultrabright Supra‐fluorophores in aqueous media, irrespective of the dye properties. Moreover, the approach has been demonstrated to be particularly effective when constructing Supra‐NIR fluorophores, characterized by exceptional brightness and outstanding photostability. It is highly anticipated that the Supra‐fluorophore approach holds significant promise for advancing fluorescence bioimaging techniques, offering a new dimension to the field of fluorescent organic nanoparticles.

## Conflict of Interest

The authors declare no conflict of interest.

## Supporting information

Supporting Information

## Data Availability

The data that support the findings of this study are available from the corresponding author upon reasonable request.
