# Supplementary material for: Supra‐Fluorophores: Ultrabright Fluorescent Supramolecular Assemblies Derived from Conventional Fluorophores in Water
Source: Adv Mater. 2024 Apr 2;36(25):2401346. doi: 10.1002/adma.202401346 (PMC11475621; doi:10.1002/adma.202401346)
Supplement: Supplementary file 1 — Supporting Information [file ADMA-36-2401346-s001.pdf]

ADVANCED MATERIALS

Supporting Information

for *Adv. Mater.*, DOI 10.1002/adma.202401346

Supra-Fluorophores: Ultrabright Fluorescent Supramolecular Assemblies Derived from Conventional Fluorophores in Water

Yuqing Lei, Yuqian Wang, Sophie K. Hill, Zihe Cheng, Qiao Song and Sébastien Perrier**

Supporting Information

Supra-Fluorophores: Ultrabright Fluorescent Supramolecular Assemblies Derived from Conventional Fluorophores in Water

Yuqing Lei, Yuqian Wang, Sophie K. Hill, Zihe Cheng, Qiao Song, and Sébastien Perrier**

S1. Materials and Characterization

Materials

Cyclic peptide (CP-NH_2) with a sequence of cyclo-(L-Lys-D-Leu-L-Trp-D-Leu-L-Trp-D-Leu-L-Trp-D-Leu-) was synthesized according to the literature.[1] PABTC, cyanine dyes (Cy3-COOH, Cy5-COOH, Cy7-COOH, ICG-COOH) were synthesized within the group. *O*-(7-azabenzotriazol-1-yl)-*N*,*N*,*N'*,*N'*-tetramethyl uronium hexafluorophosphate (HATU), *N*-methylmorpholine (NMM), and other chemicals were purchased from several suppliers, including Bidepharm, J&K, and Sigma-Aldrich. Solvents were purchased from several local suppliers, including General-Reagent, and J&K.

Characterization

Nuclear Magnetic Resonance Spectroscopy (NMR): ^{1}H NMR spectra were measured using a Bruker Avance III HD 400 MHz NMR spectrometer with chloroform-*d* ($CDCl_3$) or dimethyl sulfoxide-d_6 (DMSO-d_6) as the solvent. The residual solvent peaks were used as internal references.

Liquid Chromatography-Mass Spectrometry (LC-MS): LC-MS analysis was conducted using a WATERS H-Class/QDa Mass Spectrometer coupled with a WATERS ACQUITY UPLC to characterize the chemicals in positive ion mode. Water and acetonitrile were used as mobile

phase A and B, respectively. All solvents contained 0.1 % (v/v) formic acid. Samples were dissolved in mobile phase B with a final concentration of 0.1 mg mL^{-1} and the injection volume was 2 μL.

Matrix-Assisted Laser Desorption/Ionization-Time of Flight Mass Spectrometry (MALDI-TOF MS): MALDI-TOF MS measurements were conducted using Bruker Autoflex Speed LRF, equipped with a 355 nm Nd:YAG laser. DCTB was used as a matrix (20 mg mL^{-1} in acetonitrile).

Gel Permeation Chromatography (GPC): Molecular weights were determined by GPC measurements, which were carried out on an Agilent Infinity II MDS instrument equipped with various detectors including differential refractive index (DRI), variable wavelength ultraviolet (UV) detectors and viscometry (VS). Two PLgel Mixed D columns (300 mm × 7.5 mm) and a PLgel 5 μm guard column were included in the system calibrated using poly(methyl methacrylate) (AgilentEasiVials). The eluent of this system is DMF with 5 mmol NH_4BF_4 additive. All samples were prepared by dissolving in DMF and then filtered by 0.2 μm pore size PTFE membranes before measurement. Exported data were processed by the Agilent GPC/SEC software.

Ultraviolet–Visible (UV–Vis) Absorption Spectroscopy: UV–Vis absorption spectra were measured using a SHIMADZU UV-2600i UV–vis spectrometer. Unless otherwise stated, the path length of the cuvette was 10 mm.

Fluorescence Emission Spectroscopy: Fluorescence emission spectra were measured using either an Edinburgh Instruments FLS1000 photoluminescence spectrometer or a HITACHI F-4700 fluorescence spectrometer.

Fluorescence Quantum Yield (Φ_F): These measurements were performed using an Edinburgh FLS1000 photoluminescence spectrometer, equipped with an integrating sphere.

Time-resolved Fluorescence Spectroscopy: Fluorescence lifetime measurements were performed using an Edinburgh Instruments FLS1000 photoluminescence spectrometer, equipped with several Pulsed Lasers - EPL Series. The measurements were conducted at room temperature.

Small Angle Neutron Scattering (SANS): SANS was carried out at the SANS instrument in China Spallation Neutron Source (CSNS). The sample to detector distance was set to 4 m, with a wavelength band from 1 Å to 9.8 Å, yielding a q-range of 0.005 Å^{-1} – 0.9^{-1} Å^{-1}. Samples with deuterated solvent were loaded in the Hellma quartz cells with 2 mm light path and measured at room temperature. The scattering profiles of the samples have been calibrated to absolute scaling with sample transmission and a secondary standard sample (Bates-poly) provided by the beamline. The corresponding background contributions including solvent, empty cell as well as empty beam data were also collected and properly subtracted before data analysis.

Confocal Laser Scanning Microscopy (CLSM): The cell imaging was done using a confocal fluorescence microscope (TCS SP8, Leica; LSM 880, Zeiss). The excitation wavelengths are 405 nm (H33342), 488 nm (Lysotracker Green), and 635 nm (Cy5).

Statistical Analysis: Continuous variables are expressed as means ± standard deviation (SD) calculated from at least 3 measurements.

S2. Synthesis of Supramolecular Spacers

a. Polymer synthesis

***p*BA**: For the synthesis of the first block, PABTC, *n*-butyl acrylate, V601 initiator, and 1,4-dioxane were all weighted according to Table S1 into a glass vial with a magnetic stirrer and sealed with a rubber septum. The solution was deoxygenated by bubbling nitrogen for 10 min. The vial was then placed in an oil bath set at 70 °C. The conversion was monitored by ^{1}H NMR by taking samples every 1 h with a degassed syringe. After 4 hours of polymerization, the conversion was up to 97% and the reaction was quenched by exposure to air. The solvent was then removed under vacuum to give ***p*BA** as yellow viscous liquids.

Table S1 Synthesis of ***p*BA**.

Polymer	BA	PABTC	V601	Dioxane μL	Conversion %	$M_{n, NMR}$ g mol^{-1}	$M_{n, GPC}$ g mol^{-1}	$Đ$
pBA$_{10}$	500 mg<br>3.90 mmol	93 mg<br>0.39 mmol	2.3 mg<br>9.8 μmol	517	99.2	1520	1100	1.07
pBA$_{20}$	1000 mg<br>7.80 mmol	93 mg<br>0.39 mmol	2.3 mg<br>9.8 μmol	1258	97.5	2800	1700	1.07
pBA$_{30}$	1000 mg	62 mg	1.5 mg	1332	98.5	4080	2200	1.10

	7.80 mmol	0.26 mmol	6.5 μmol

***p*DMA$_{60}$ (P1)**: For the synthesis of *p*DMA homopolymer, PABTC (40.1 mg, 0.17 mmol), DMA (1 g, 10.09 mmol), V601 initiator (1.2 mg, 5.04 μmol), and 1,4-dioxane (2.2 mL) were all weighted into a glass vial with a magnetic stirrer and sealed with a rubber septum. The solution was deoxygenated by bubbling nitrogen for 10 min. The vial was then placed in an oil bath set at 70 °C. The conversion was monitored by ^{1}H NMR by taking samples every 1 h with a degassed syringe. After 3 h of polymerization, the conversion was 95.9% and the reaction was quenched by exposure to air. The dioxane solution was then precipitated in diethyl ether (twice) and dried under vacuum to give ***p*DMA$_{60}$ (P1)** as a yellow solid. Yield = 86.4% (898 mg). $M_{n, NMR}$ = 6190 g mol^{-1}, $M_{n, GPC}$ = 7200 g mol^{-1}, $Đ$=1.14.

***p*BA-*b*-*p*DMA (P2-P4)**: For the synthesis of *p*BA-*b*-*p*DMA deblock copolymer, *p*BA was used as a Macro-CTA. Macro-CTA, DMA, V601 initiator, and 1,4-dioxane were all weighted according to Table S2 into a glass vial with a magnetic stirrer and sealed with a rubber septum. The solution was deoxygenated by bubbling nitrogen for 10 min. The vial was then placed in an oil bath set at 70 °C. The conversion was monitored by ^{1}H NMR by taking samples every 1 h with a degassed syringe. After 3 h of polymerization, the conversion was up to 97% and the reaction was quenched by exposure to air. The dioxane solution was then precipitated in diethyl ether (twice) and dried under vacuum to give ***p*BA-*b*-*p*DMA (P2-P4)** as yellow solids.

Table S2 Synthesis of **P2-P4**.

Polymer	DMA	Macro-CTA	V601	Dioxane μL	Conversion %	$M_{n, NMR}$ g mol^{-1}	$M_{n, GPC}$ g mol^{-1}	$Đ$
**P2**	1000 mg<br>10.09 mmol	212 mg<br>0.16 mmol	1.2 mg<br>5.0 μmol	2207	97.9	7470	9800	1.15
**P3**	1000 mg<br>10.09 mmol	385 mg<br>0.16 mmol	1.2 mg<br>5.0 μmol	2207	98.9	8750	10100	1.14
**P4**	1000 mg<br>10.09 mmol	622 mg<br>0.16 mmol	1.2 mg<br>5.0 μmol	2207	99.9	10030	11000	1.14

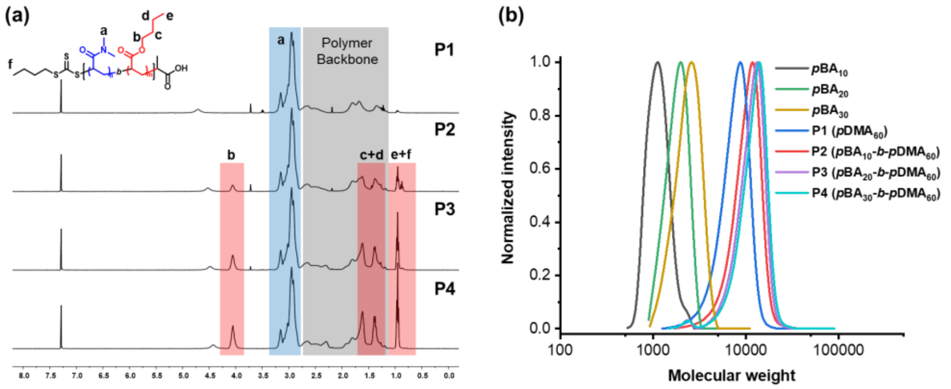

Figure S1 Characterization of **P1-P4**. (a) ^{1}H NMR spectra of **P1-P4** ($CDCl_3$, 400 MHz); (b) GPC traces of ***p*BA** and **P1-P4**.

b. Conjugates S1-S4 synthesis

To a 1 mL DMF solution of CP-NH_2 (10 mg, 8.8 μmol), **P1-P4** (13.2 μmol), HATU (5.0 mg, 13.2 μmol) and NMM (2.7 mg, 26.3 μmol) were added. The reaction was left for 24 h. The DMF solution was then precipitated in a mixed solvent of dichloromethane and diethyl ether to remove unreacted polymers (twice) and dried under vacuum to obtain **S1-S4** as yellow solids.

Table S3 Synthesis of **S1-S4**.

Conjugate	CP-NH_2	Polymer	DCM/Ether	Yield / %
**S1**	10 mg<br>8.8 μmol	**P1**<br>81.4 mg<br>13.2 μmol	1/3	48%
**S2**	10 mg<br>8.8 μmol	**P2**<br>94.9 mg<br>13.2 μmol	1/7	57%
**S3**	10 mg<br>8.8 μmol	**P3**<br>108.4 mg<br>13.2 μmol	1/6	79%
**S4**	10 mg<br>8.8 μmol	**P4**<br>127.0 mg<br>13.2 μmol	0/1	82%

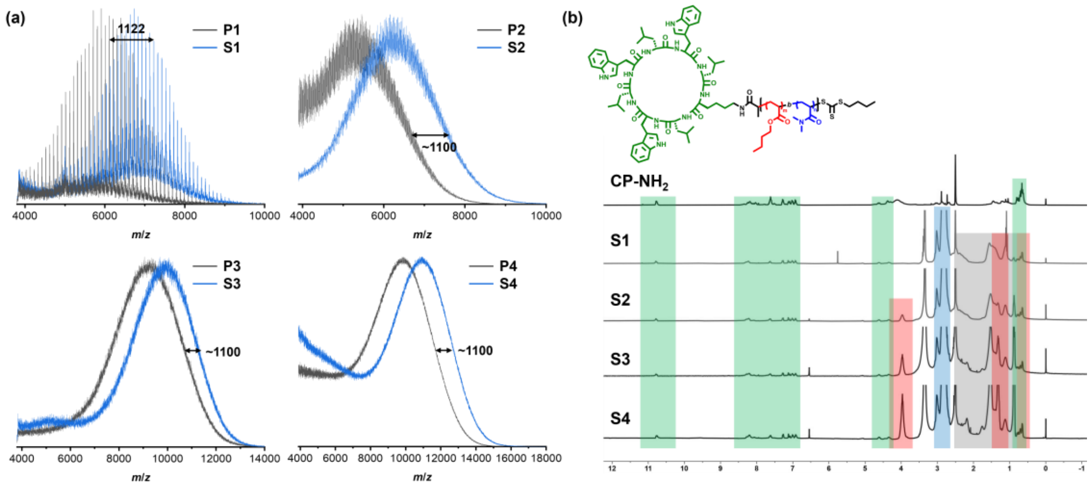

Figure S2 Characterization of **S1-S4**. (a) MALDI-TOF MS of **P1-P4** and **S1-S4**; (b) ^{1}H NMR spectra of **S1-S4** (400 MHz, DMSO-d_6).

S3. Self-assembly characterization

The self-assembly of the supramolecular spacers **S1-S4** was realized by firstly dissolving it in a small amount of DMF, followed by the addition of DI water, resulting clear solutions with H_2O/DMF ratio of 95/5. In the case of SANS, deuterated solvents were used (i.e. DMSO-d_6 instead of DMF, D_2O instead of H_2O).

For the co-assembly of **S1-S4** and CP-Fluorophores (preparation of Supra-fluorophores), conjugates were premixed at certain molar ratios in DMF before adding DI water to obtain solutions with desired concentrations while keeping H_2O/DMF ratio as 95/5. The solutions were kept in the dark at room temperature before measurements.

For Supra-fluorophores in the solid states, the aqueous solution of Supra-fluorophore was drop-casted on a quartz substrate, which was pretreated by a UV-ozone-cleaning procedure. The quartz substrate was then allowed to air-dry for a duration of 24 h in a dark and well-ventilated environment.

SasView software was used to fit the SANS data, using a core-shell cylinder model. In this case, the core corresponds to the cyclic peptide, and the shell is assumed to be solvated polymer. SLD values were calculated based on the molecular structure of the conjugate and solvent. The radius of the core value was fixed at 5 Å, representing the radius of the cyclic peptide itself. The fitting procedure was performed to minimize the reduced χ^2, which is normalized by the number of

data points and the number of fitting parameters.

Table S4 Fitting parameters using a core-shell cylinder model implemented with SasView.

SAMPLE	**S1**	**S2**	**S3**	**S4**
Scale	0.825±0.043	0.263±0.023	0.267±0.018	0.173±0.019
Background*	0.0015	0.0035	0.0022	0.0022
sld_core*	2.03	2.03	2.03	2.03
sld_shell	6.243±0.003	6.169±0.010	6.091±0.011	5.973±0.023
sld_solvent*	6.376	6.376	6.376	6.376
Radius*	5	5	5	5
Thickness	40.1±0.1	39.3±0.2	42.3±0.1	49.3±0.1
Length	42.4±2.0	46.6±2.1	74.7±1.3	65.5±1.0
Distri. of thickness*	0.3	0.2	0.2	0.1
Reduced χ^2	3.94	1.50	2.29	3.25

Parameters marked with * were held constant throughout the fitting procedure, mean ± SD.

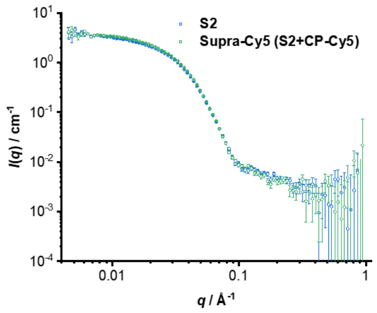

Figure S3 Comparation of the SANS scattering data of **S2** and Supra-Cy5 (**S2**+CP-Cy5).

S4. Photophysical Performance of Supra-Cy5 Built by Different Spacers

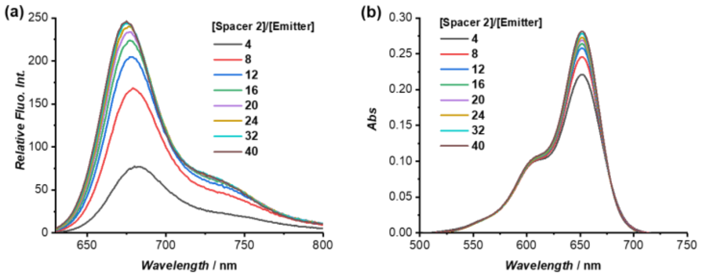

Figure S4 Fluorescence spectra (a) and UV/vis absorption spectra (b) of Supra-Cy5 constructed by CP-Cy5 and **S2** at higher molar ratios ([CP-Cy5]=2 μM, water/DMF=95/5,

λ_{ex}=620 nm).

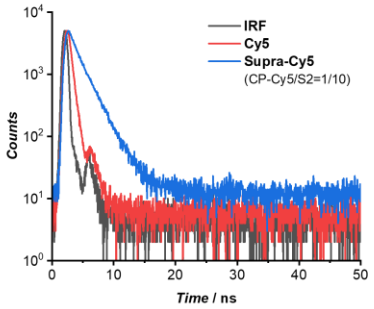

Figure S5 Fluorescence decay profiles of Cy5 and Supra-Cy5.

Table S5 Photophysical properties of Supra-Cy5 and Cy5 in water.

	Φ_F / %	τ_1 / ns	τ_2 / ns	$<\tau>$ / ns	k_r / 10^8 s^{-1}	k_{nr} / 10^8 s^{-1}
Cy5	13.2	0.47 (100%)	-	0.47	2.81	18.47
Supra-Cy5 (S2/CP-Cy5=10/1)	30.7	0.65 (15.0%)	2.18 (85.0%)	1.61	1.91	4.31

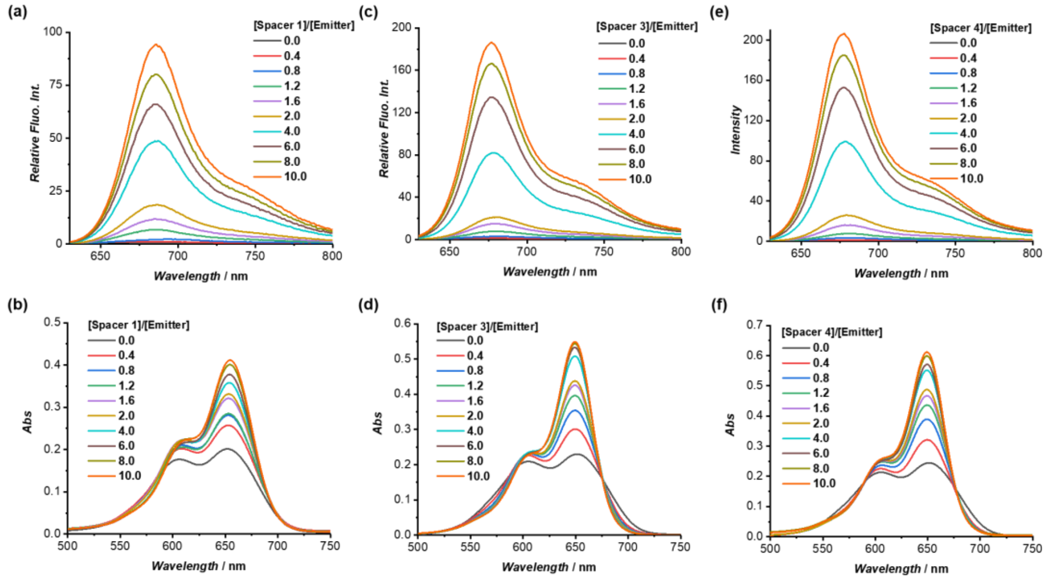

Figure S6 Fluorescence spectra and UV/vis absorption spectra of Supra-Cy5 constructed by CP-Cy5 and **S1** (a, b), **S3** (c, d), and **S4** (e, f) at different molar ratios ([CP-Cy5]=4 μM, water/DMF=95/5, λ_{ex}=620 nm).

Table S6 Summary of fluorescence quantum yield (Φ_F) and brightness (B) of Supra-Cy5 using S1-S4 as Spacer at different Spacer/Emitter molar ratios.

	**S1**	**S2**	**S3**	**S4**

[Spacer]/ [Emitter]	Φ_F %	B $M^{-1}cm^{-1}$	Φ_F %	B $M^{-1}cm^{-1}$	Φ_F %	B $M^{-1}cm^{-1}$	Φ_F %	B $M^{-1}cm^{-1}$
1.2	-	-	2.4±0.6	1950±490	1.6±0.1	1590±100	2.4±0.2	2420±150
1.6	2.3±0.1	1840±80	3.6±0.8	3170±670	3.2±0.1	3410±110	3.3±0.8	3640±880
2.0	2.1±1.5	1740±1240	4.8±0.9	4290±850	5.3±1.2	5750±1340	4.6±0.9	5230±1010
4.0	6.4±2.4	5730±2150	12.7±1.6	13970±1760	14.3±2.4	18100±2990	14.2±1.6	18410±2020
6.0	9.0±2.6	8500±2450	19.7±1.0	23940±1220	22.3±2.8	30460±3670	21.7±2.2	29250±2970
8.0	12.8±1.6	12830±1600	26.3±1.5	32770±1870	29.5±3.7	40050±4970	26.2±1.9	37020±2680
10.0	16.7±3.0	17200±3100	30.7±1.3	37980±1570	33.6±5.7	46060±7870	29.9±2.3	43150±3270

*[CP-Cy5]=4 μM, water/DMF=95/5. The values represent the mean value (and standard deviation) of at least 3 measurements.

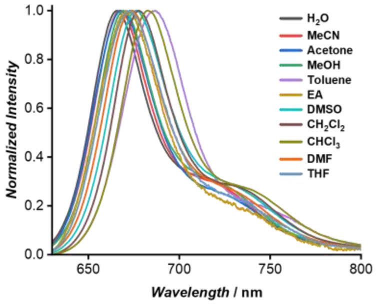

Figure S7 Normalized fluorescence spectra of Cy5 (4 μM) in different solvents.

As shown in Figure S7, distinct variations were observed in its emission maximum across different solvents. Thus, it is reasonable to attribute the bathochromic shift of Cy5 within the assemblies to changes in the microenvironment. However, the relationship between the emission maximum and solvent polarity does not follow a specific trend. Therefore, NMI is used to probe the polarity of the hydrophobic inner region within these assemblies.

Determination of the polarity using NMI as probe: NMI was dissolved in the solvents shown in Figure S6a at a centration of 4 μM, respectively. The emission spectra of the NTI solutions were then measured (Figure S8a). The emission maximum was plotted against the logarithm of the solvent's dielectric constant, and a linear correlation was conducted (Figure S8b), giving an equation as follows:

$$y = 26.7x + 447.8 \qquad \text{Eq. S1}$$

Where x is the logarithm of the solvent's dielectric constant, y is the emission maximum of NMI. Then CP-NMI was co-assembled with **S1-S4** in a molar ratio of 1/10 ([CP-NMI]= 4 μM),

and the emission spectra of the resulting 4 solutions were measured (Figure S8c). The emission maximum was used to calculate the polarity (dielectric constant) of the inner region within the assemblies according to Eq. S1.

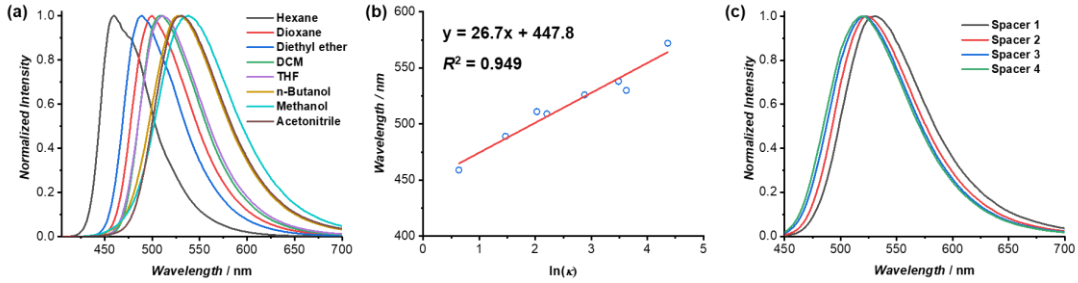

Figure S8 (a) Fluorescence spectra of NMI in various solvents; (b) Plot and linear fitting of the emission maximum of NMI against the logarithm of the solvent's dielectric constant; (c) Normalized fluorescence spectra of CP-NMI assembled with **S1-S4**.

Determination of the viscosity using CCVJ as probe: CCVJ was dissolved in a mixed solvent of ethylene glycol and glycerol at different volume ratios shown in Figure S7 at a centration of 4 μM, respectively. The emission spectra of the CCVJ solutions were then measured (Figure S9a). The emission intensity was plotted against the solvent's viscosity, and a linear correlation was found in a double logarithmic scale (Figure S9b), giving an equation as follows:

$$y = 0.59x + 1.46 \qquad \text{Eq. S2}$$

Where x is the logarithm of the solvent's viscosity, y is the logarithm of the emission intensity of CCVJ. Then CP-CCVJ was co-assembled with **S1-S4** in a molar ratio of 1/10 ([CP-CCVJ]= 4 μM), and the emission spectra of the resulting 4 solutions were measured (Figure S9c). The emission intensity was used to calculate the viscosity of the inner region within the assemblies according to Eq. S2.

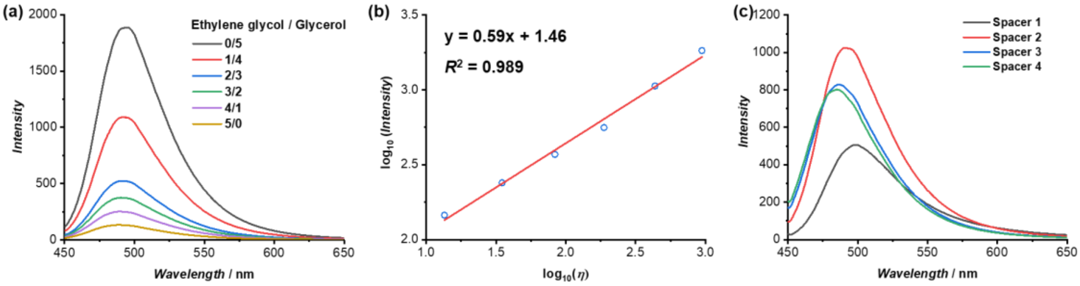

Figure S9 (a) Fluorescence spectra of CCVJ in a mixed solvent of ethylene glycol and

glycerol at different volume ratios; (b) Double logarithmic plot and linear fitting of the emission intensity of CCVJ against the solvent's viscosity; (c) Fluorescence spectra of CP-CCVJ assembled with **S1-S4**.

The two probes, NMI and CCVJ, are recognized as twisted intramolecular charge transfer (TICT) dyes, whose luminescent capability is sensitive to their microenvironment. Considering the densely packed surroundings within the supramolecular scaffold, the TICT process is expected to be suppressed. As evidence, the fluorescence quantum yields of NMI and CCVJ within the assemblies were measured to be 12.3% and 5.8% respectively, while the values of free NMI and CCVJ in water were far below 1%.

Volume-normalized Brightness of Supra-Cy5

Calculation of B_V and local concentration of Cy5: The average volume *per* dye occupies (V) is calculated by assuming CP-Cy5 is evenly separated by Spacer. Considering that the distance between two cyclic peptides is 0.47 nm, and the diameter of the cyclic peptide is 1 nm, the relationship between V and the molar ratio of Spacer/Emitter (n) could be estimated as:

$$V = (n + 1) * 0.47 * \pi * 0.5^2 \ nm^3 \qquad \text{[Eq. S3]}$$

Hence, the volume-normalized brightness B_V follows the equation shown below:

$$B_V = \frac{\Phi_F * \varepsilon}{(n+1)*0.47*\pi*0.5^2} \ \mathrm{M^{-1}\ cm^{-1}\ nm^{-3}} \qquad \text{[Eq. S4]}$$

The local concentration of Cy5 ($[\mathrm{Cy5}]_{local}$) could be written as:

$$[Cy5]_{local} = \frac{10^{27}}{N_A * V} \ mM, \text{ where } N_A \text{ is Avogadro constant.} \qquad \text{[Eq. S5]}$$

Table S7 Summary of photophysical properties of Supra-Cy5 using **S3** as Spacer at different Spacer/Emitter molar ratios.

[Spacer]/[CP-Cy5]	Φ_F %	ε M^{-1} cm^{-1}	B $M^{-1}cm^{-1}$	V nm^{-3}	B_V $M^{-1}cm^{-1}nm^{-3}$	$[Cy5]_{local}$ mM
1.2	1.6±0.1	99100	1590±100	0.81	1960±120	2050
1.6	3.2±0.1	106500	3410±110	0.96	3550±110	1730
2	5.3±1.2	109500	5750±1340	1.11	5180±1210	1500
4	14.3±2.4	127100	18100±2990	1.84	9840±1620	900
6	22.3±2.8	133300	30460±3670	2.58	11810±1420	640

8	29.5±3.7	136000	40050±4970	3.32	12060±1500	500
10	33.6±5.7	137300	46060±7870	4.06	11350±1940	410

*[CP-Cy5]=4 μM, water/DMF=95/5. The values represent the mean value (and standard deviation) of at least 3 measurements.

Preparation of Supra-Cy5 in different aqueous media. A DMF stock solution containing **S3** (800 μM) and CP-Cy5 (80 μM) was first prepared. Then 100 μL of the DMF solution was taken to mix with 1900 μL of water, PBS, Tris, DMEM cell culture media, fetal bovine serum (FBS), and DMEM+10%FBS, respectively, resulting 2 mL of Supra-Cy5 (4 μM, [**S3**]/[CP-Cy5]=10/1) solutions in different media. The Supra-Cy5 solutions were left in the dark at room temperature for at least 24 h before fluorescence measurements. The measurements were conducted at 25 °C.

S5. Cell imaging

50,000 CT26 Colorectal Cancer cells were seeded in each well of an 8 well chamber and incubated overnight at 37°C/5%CO_2. The following day, the cells were washed with PBS and 250 μL of each prepared compound (Supra-Cy5 or Cy5, 5 μM) in DMEM was added, before returning cells for incubation for 24 h. The cells were washed twice with PBS and then phenol red free DMEM was added. The imaging of the cells was performed using CLSM. For the intracellular localization experiment, Hela cells were used, which were incubated with Supra-Cy5 (2 μM) for 2 hours at 37°C, followed by thorough washing with PBS. After incubating the Hela cells with Hoechst 33342, and LysoTracker Green, the imaging of the cells was performed using CLSM.

S6. Expansion to 6 Categories of Organic Dyes

Table S8 Summary of photophysical properties of Supra-fluorophores (1st line) and free fluorophores (2nd line) in aqueous solutions

Dye	λ_{abs} nm	ε M^{-1} cm^{-1}	λ_{em} nm	Φ_F %	B $M^{-1}cm^{-1}$	B_V $M^{-1}cm^{-1}nm^{-3}$
DPA	375	14300	432	30.8±4.5	4400±640	2320±440 (2/1)
	375	13590	434	13.9±0.4	1890±50	n/a

Cou343	440	29960	481	28.8±4.7	8630±1410	2870±500 (2/1)
	427	27500	490	100	27500	n/a
BDP_FL	508	80320	517	22.6±2.8	18150±2250	4460±410 (10/1)
	503	71290	512	97.8±2.2	69720±160	n/a
Cy3	552	91050	571	22.5±0.3	20490±270	6400±300 (6/1)
	542	112550	560	2.8±0.1	3150±110	n/a
RhB	569	76100	592	17.1±0.3	13010±230	5210±360 (2/1)
	565	84490	581	31.6±1.1	26700±930	n/a
SQ	638	159140	655	21.8±1.1	34690±1750	8580±660 (10/1)
	622	124450	636	2.4±0.5	2990±620	n/a

*[CP-Fluorophores]=4 μM, water/DMF=95/5. The values represent the mean value (and standard deviation) of at least 3 measurements.

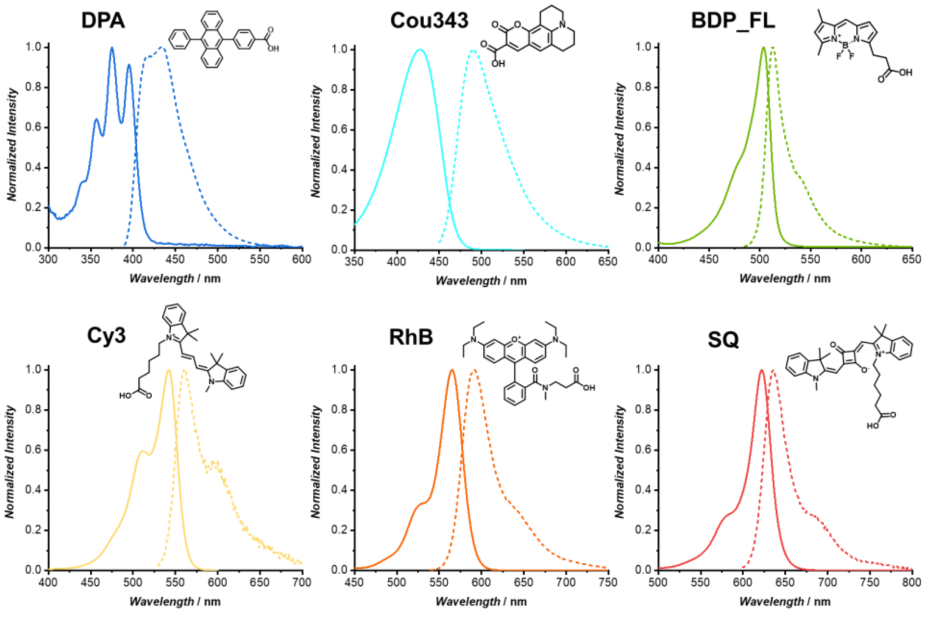

Figure S10 Normalized UV/Vis absorption spectra and fluorescence spectra of DPA, Cou343, BDP_FL, Cy3, RhB, and SQ in water (4 μM).

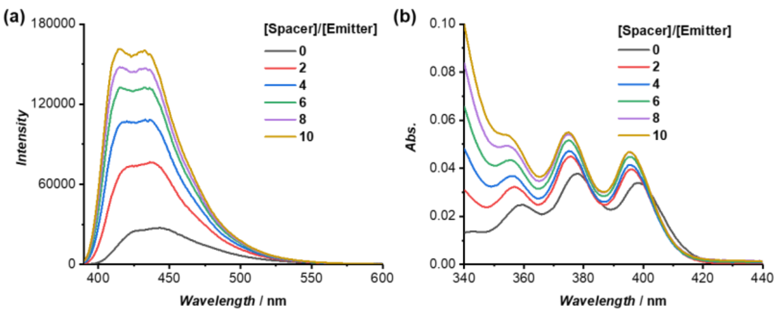

Figure S11 Fluorescence spectra (a) and UV/Vis absorption spectra (b) of Supra-DPA constructed by CP-DPA and **S3** at different molar ratios ([CP-DPA]=4 μM, water/DMF=95/5,

λ_{ex}=370 nm).

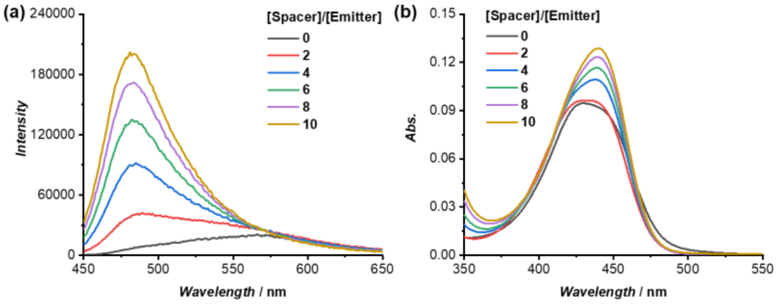

Figure S12 Fluorescence spectra (a) and UV/vis absorption spectra (b) of Supra-Cou343 constructed by CP-Cou343 and **S3** at different molar ratios ([CP-Cou343]=4 µM, water/DMF=95/5, λ_{ex}=440 nm).

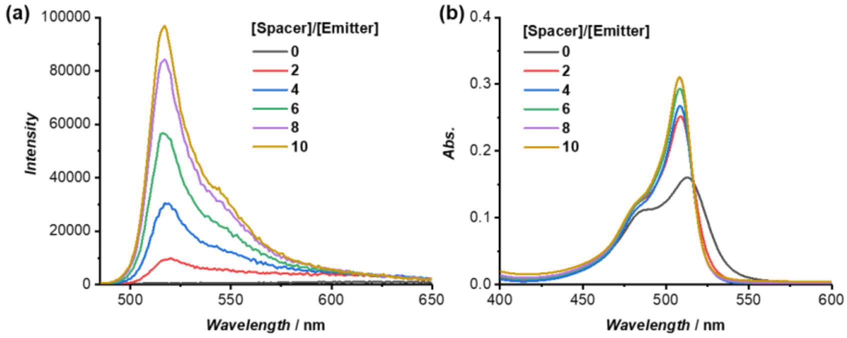

Figure S13 Fluorescence spectra (a) and UV/vis absorption spectra (b) of Supra-BDP_FL constructed by CP-BDP_FL and **S3** at different molar ratios ([CP-BDP_FL]=4 µM, water/DMF=95/5, λ_{ex}=480 nm).

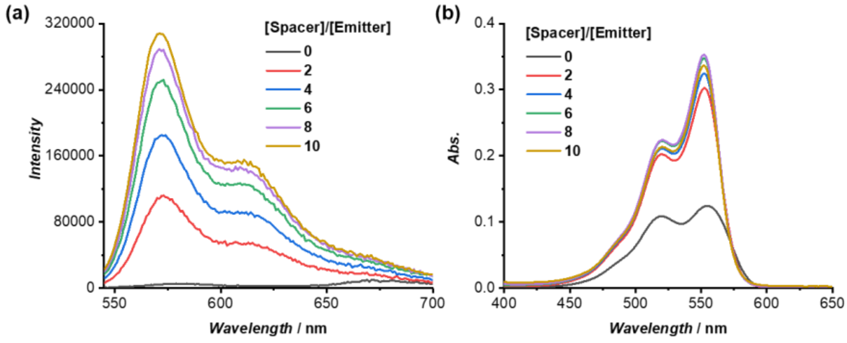

Figure S14 Fluorescence spectra (a) and UV/vis absorption spectra (b) of Supra-Cy3 constructed by CP-Cy3 and **S3** at different molar ratios ([CP-Cy3]=4 µM, water/DMF=95/5, λ_{ex}=520 nm).

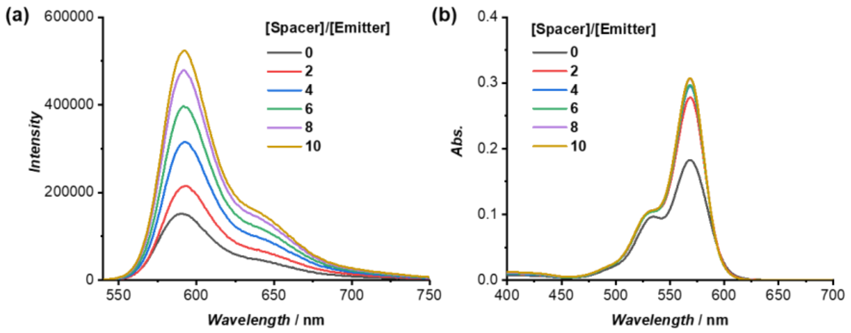

Figure S15 Fluorescence spectra (a) and UV/vis absorption spectra (b) of Supra-RhB constructed by CP-RhB and **S3** at different molar ratios ([CP-RhB]=4 μM, water/DMF=95/5, λ_{ex}=500 nm).

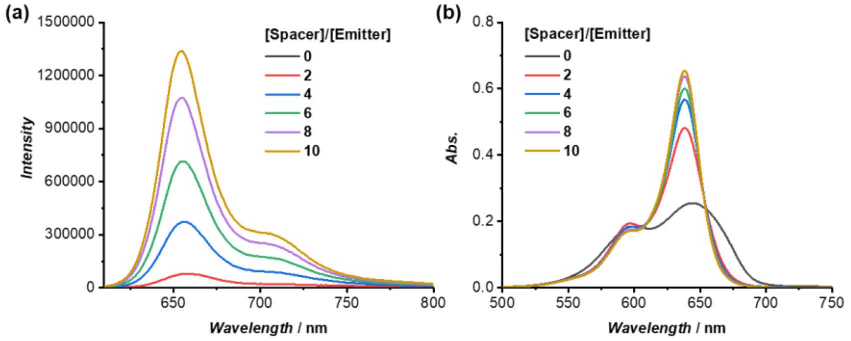

Figure S16 Fluorescence spectra (a) and UV/vis absorption spectra (b) of Supra-SQ constructed by CP-SQ and **S3** at different molar ratios ([CP-SQ]=4 μM, water/DMF=95/5, λ_{ex}=595 nm).

Table S9 Summary of photophysical properties of Supra-fluorophores in solution and solid states.

		λ_{abs} / nm	λ_{em} / nm	Φ_F / %
**DPA**	Solution	375	432	30.8
	Film	375	437	12.7
**Cou343**	Solution	440	481	28.8
	Film	441	467	20.1
**BDP_FL**	Solution	508	517	22.6
	Film	509	517	49.9
**Cy3**	Solution	552	571	22.5
	Film	555	573	37.3
**RhB**	Solution	569	592	17.1
	Film	566	588	42.8

**Cy5**	Solution	649	677	33.6
	Film	650	677	26.8
**SQ**	Solution	638	655	21.8
	Film	640	654	9.6

Fluorescent Printing Using the Supra-fluorophores as Inks: The black and tricolor ink cartridges (HP 805) of a commercial inkjet printer (HP Deskjet 1212) were washed extensively with deionized water. Aqueous solutions of Supra-DPA, Supra-BDP_FL, Supra-Cy3, and Supra-SQ (50 μM) were filled into the empty black and tricolor ink cartridges by syringes. Subsequently, fluorescent patterns were printed on white papers that showed no background UV fluorescence. Fluorescent images of multicolor patterns were obtained using a cell phone under a 310 nm UV lamp.

S7. Supra-NIR Dyes with High Brightness and Outstanding Photostability

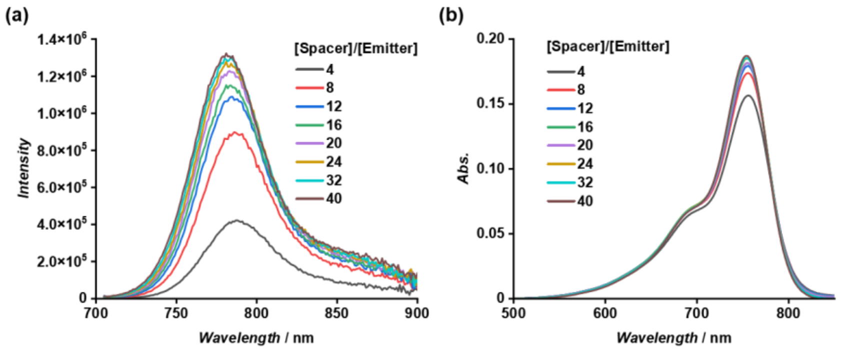

Figure S17 Fluorescence spectra (a) and UV/Vis absorption spectra (b) of Supra-Cy7 constructed by CP-Cy7 and **S3** at different molar ratios ([CP-Cy7]=2 μM, water/DMF=95/5, λ_{ex}=700 nm).

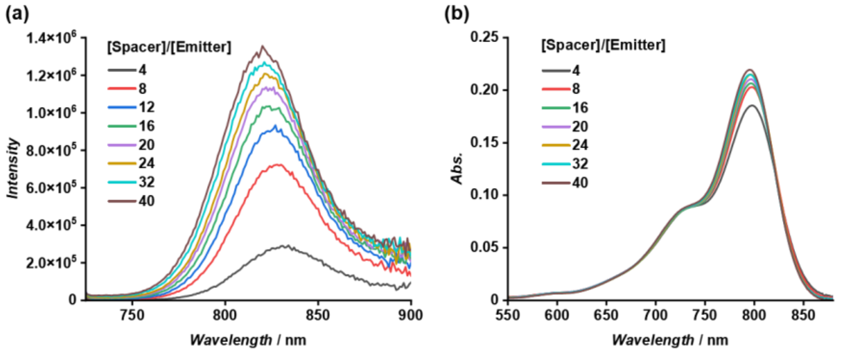

Figure S18 Fluorescence spectra (a) and UV/Vis absorption spectra (b) of Supra-ICG constructed by CP-ICG and **S3** at different molar ratios ([CP-ICG]=2 μM, water/DMF=95/5,

λ_{ex}=720 nm).

Photostability: A total of 2 mL of Supra-Cy7 or Supra-ICG solution (2 μM, with a [Spacer]/[Emitter] ratio of 20/1) and a separate 2 mL of Cy7 or ICG solution (2 μM) were subjected to UV-Vis absorption spectrometry measurements at 1-min intervals over a 30-min time period. These measurements were conducted while exposing the solutions to irradiation from a Xenon (Xe) lamp. The absorption spectra were normalized with respect to the spectrum obtained before irradiation, and the absorption maximum was then plotted as a function of irradiation time. The data was fitted according to Eq. S6 to obtain the rate constants of the photodegradation reaction.

$$y = e^{-kx} \quad \text{[Eq. S6]}$$

Where x is irradiation time, y is the relative absorption maximum of the dye, k is the rate constant of the photodegradation reaction.

S8. Synthesis of Supramolecular Emitters

a. Synthesis of RhB-COOH

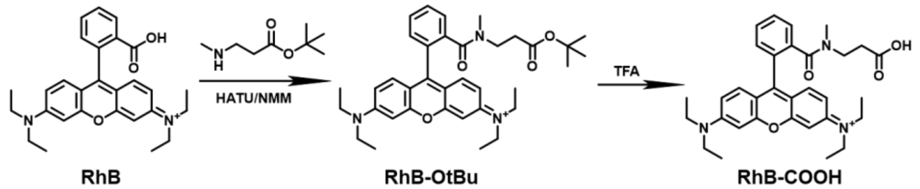

(1) Synthesis of RhB-OtBu

To a solution of RhB (643 mg, 1.45 mmol) in DMF (20 mL), TEA (441 mg, 4.36 mmol) and HATU (597 mg, 1.57 mmol) were added. After 10 min, *tert*-butyl 3-(methylamino)propanoate (250 mg, 1.57 mmol) was added and the reaction mixture was stirred at room temperature for 2 h. 100 mL ethyl acetate was added into the reaction flask, followed by washing with water (100 mL, twice) and brine (100 mL). The organic layer was dried over $MgSO_4$ and evaporated to remove the solvent. The product **RhB-OtBu** was obtained by column chromatography on silica gel using DCM/MeOH=40/3 as a red solid (292 mg, 33%).

^{1}H NMR (400 MHz, Chloroform-*d*) δ 7.67 – 7.63 (m, 2H), 7.55 – 7.51 (m, 1H), 7.34 (d, 1H), 7.28 (s, 2H), 6.95 (dd, 2H), 6.76 (d, 2H), 3.64 – 3.57 (m, 8H), 3.41 (t, 2H), 2.95 (s, 3H), 2.12 (t, 2H), 1.36 (s, 9H), 1.33 (d, 12H).

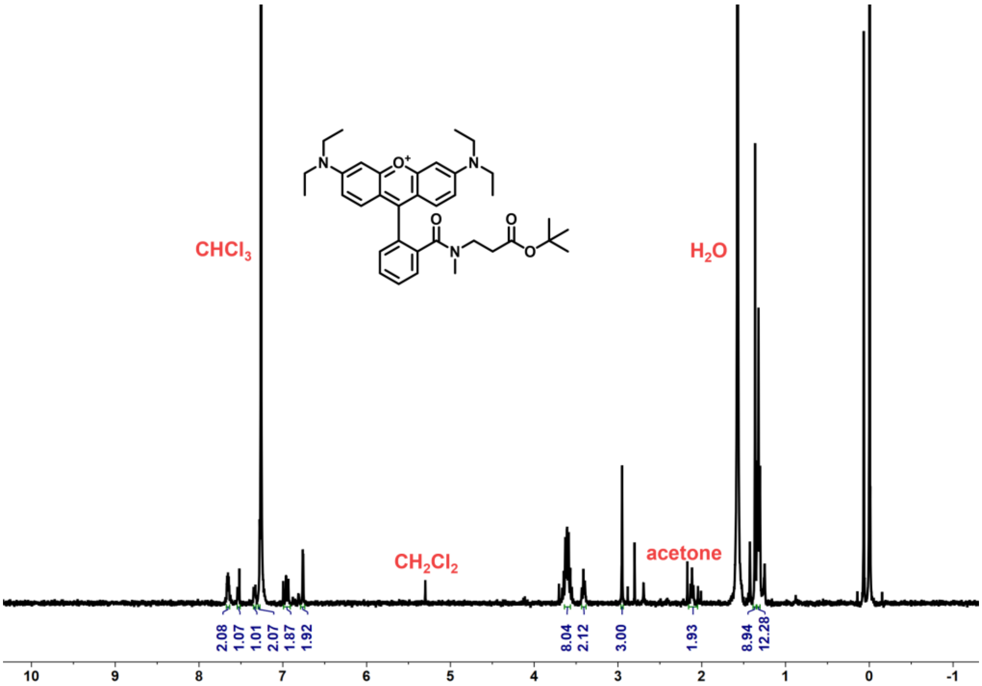

Figure S19. ^{1}H NMR (400 MHz, Chloroform-*d*) spectrum of **RhB-OtBu**.

(2) Synthesis of RhB-COOH

To a solution of **RhB-OtBu** (50 mg) in DCM (2 mL), TFA (2 mL) was added, and the reaction mixture was stirred at room temperature for 12 h. After the reaction finished, all solvents were evaporated. The resulting solid was washed with a mixed solvent of hexane and diethyl ether (3/2) twice and dried under vacuum to obtain **RhB-COOH** as a red solid (30.2 mg, 67%).

^{1}H NMR (400 MHz, Chloroform-*d*) δ 7.65 (dt, 3H), 7.35 (d, 1H), 7.27 (s, 1H), 7.25 (s, 1H), 7.03 (d, 2H), 6.82 (dd, 2H), 3.62 (q, 8H), 3.35 (t, 2H), 2.75 (s, 3H), 2.00 (t, 2H), 1.32 (t, 12H). $[M]^{+}$ calcd. for $C_{32}H_{38}N_3O_4^{+}$, m/z 528.3; found m/z 528.2.

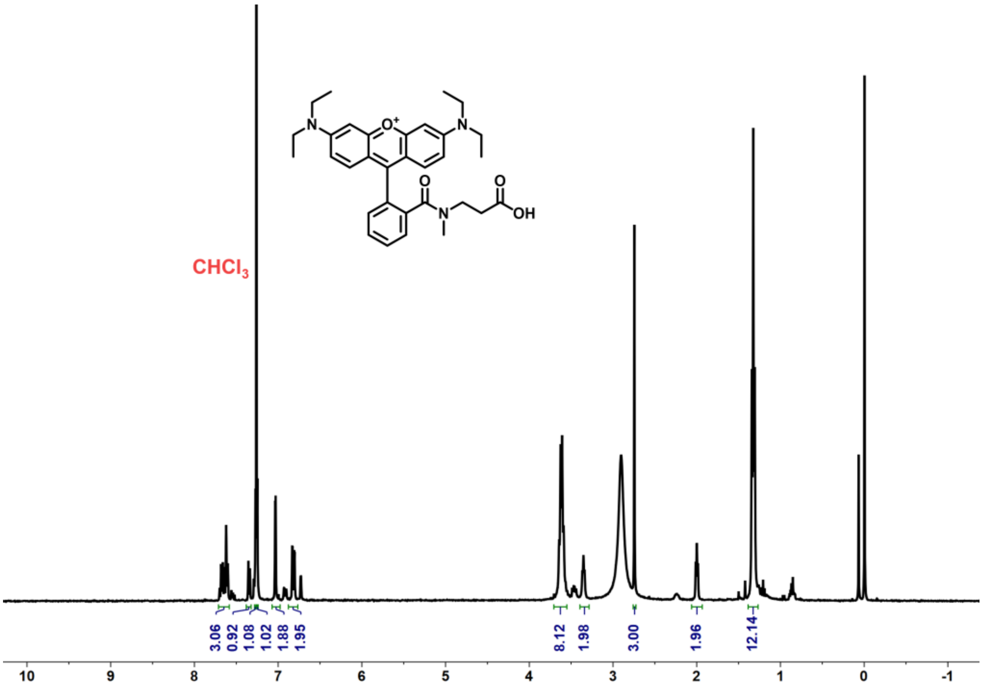

Figure S20. ^{1}H NMR (400 MHz, Chloroform-*d*) spectrum of **RhB-COOH**.

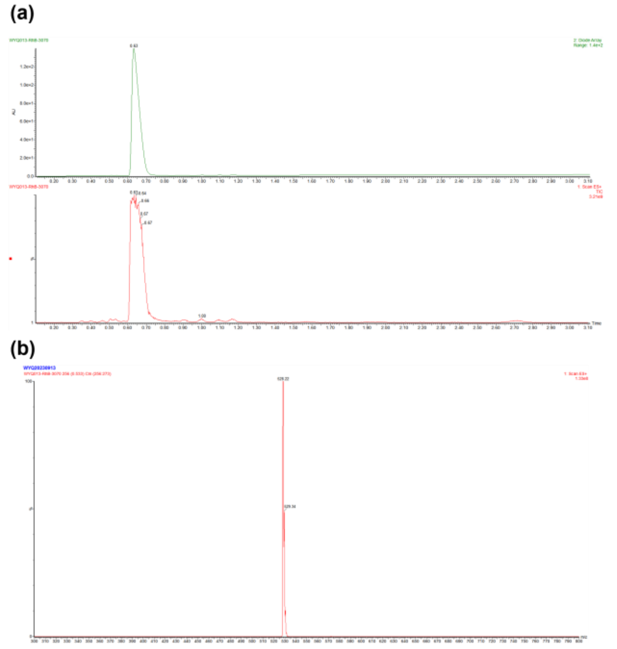

Figure S21. LC-MS of **RhB-COOH**.

b. Synthesis of SQ-COOH

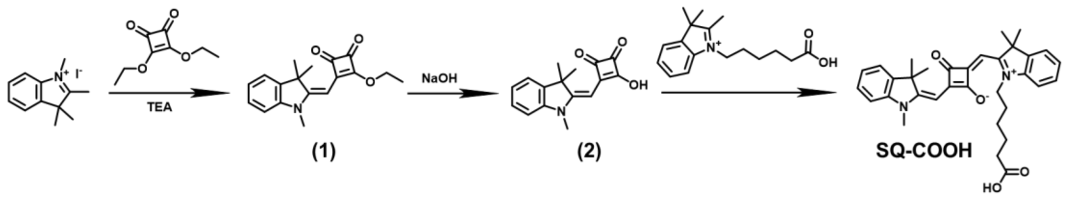

The synthesis of SQ-COOH was modified according to literature.[2]

(1) Synthesis of Intermediate 1

To a 100 mL round flask was added 1,2,3,3-tetramethyl-3*H*-indol-1-ium iodide (1.28 g, 4.25 mmol), 3,4-diethoxycyclobut-3-ene-1,2-dione (0.89 g, 5.25 mmol), and ethanol (20 mL) as the solvent. After the addition of TEA (0.7 mL, 5 mmol), the solution was heated to reflux for 15 min. The color of the solution turned to olive green. The crude product was further purified by column chromatography on silica gel using DCM/MeOH=20/1 as the eluent. The product **1** was obtained as an orange solid (0.95 g, 75%).

^{1}H NMR (400 MHz, Chloroform-*d*) δ 7.31 – 7.26 (m, 2H), 7.07 (t, 1H), 6.89 (d, 1H), 5.36 (s, 1H), 4.89 (t, 2H), 3.37 (s, 3H), 1.63 (s, 6H), 1.53 (t, 3H).

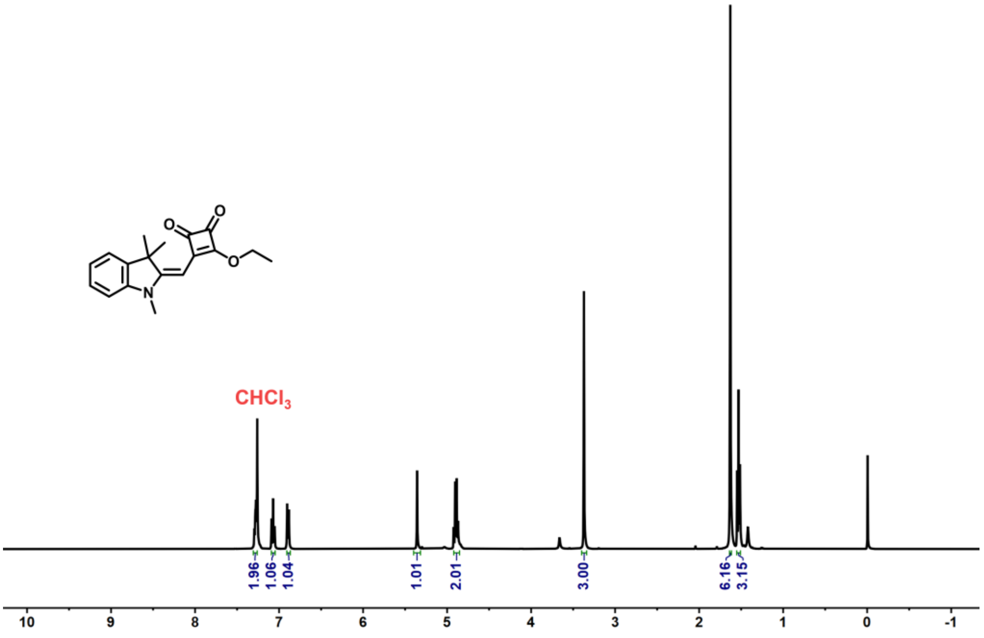

Figure S22. ^{1}H NMR (400 MHz, Chloroform-*d*) spectrum of Intermediate **1**.

(2) Synthesis of SQ-COOH

To a 50 mL round flask was added 1 (881 mg, 2.83 mmol), 40% NaOH solution (1 mL), and ethanol (10 mL) as the solvent. The solution was heated to reflux for 5 min, and then cooled to room temperature. After the solvent was evaporated, 5 mL cold ethanol was added to obtain some yellow precipitate. The solid **(2)** was washed three times with cold ethanol and dissolved in a mixture of *n*-butanol (25 mL) and toluene (25 mL). To the solution, 1-(5-carboxypentyl)-2,3,3-trimethyl-3*H*-indol-1-ium (1002 mg, 2.83 mmol) was added and the reaction mixture was refluxed for 18 h. The solvents were removed under vacuum. The residue was purified by column chromatography on silica gel using DCM/MeOH=20/1 as the eluent. The product SQ-

COOH was obtained as a blue solid (75 mg, 5%).

^{1}H NMR (400 MHz, DMSO-d_6) δ 7.51 (d, 2H), 7.33 (dd, 4H), 7.16 (t, 2H), 5.80 – 5.74 (m, 2H), 4.07 (s, 2H), 3.57 (s, 3H), 2.21 (t, 2H), 1.68 (s, 12H), 1.57 – 1.52 (m, 2H), 1.39 (s, 2H), 1.24 (d, 2H).

$[M]^+$ calcd. for $C_{33}H_{36}N_2O_4^+$, m/z 524.27; found m/z 524.28.

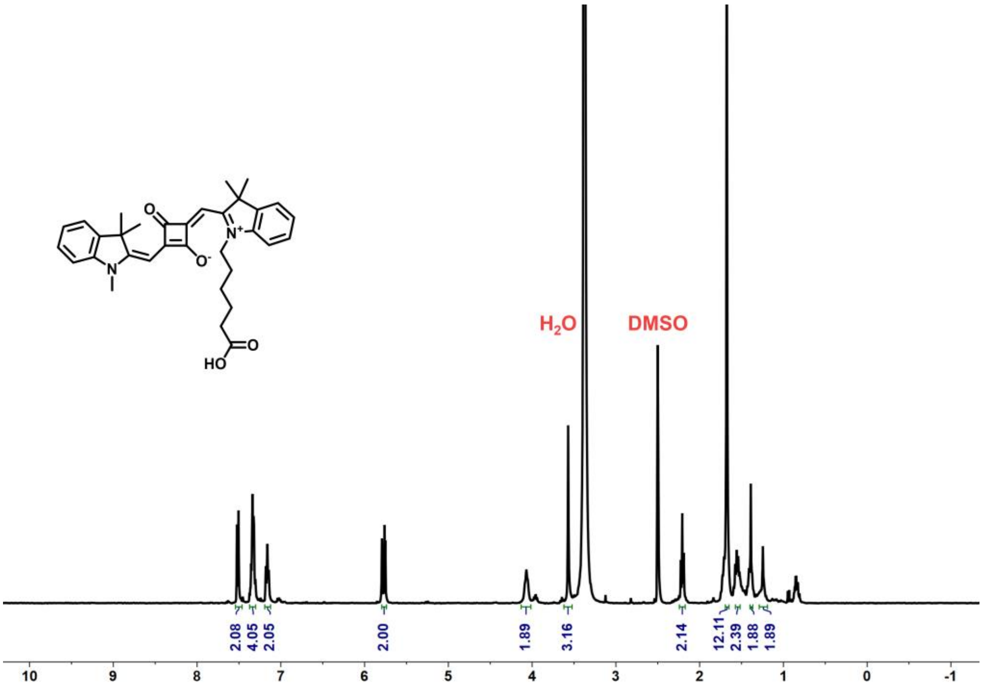

Figure S23. ^{1}H NMR (400 MHz, DMSO-d_6) spectrum of **SQ-COOH**.

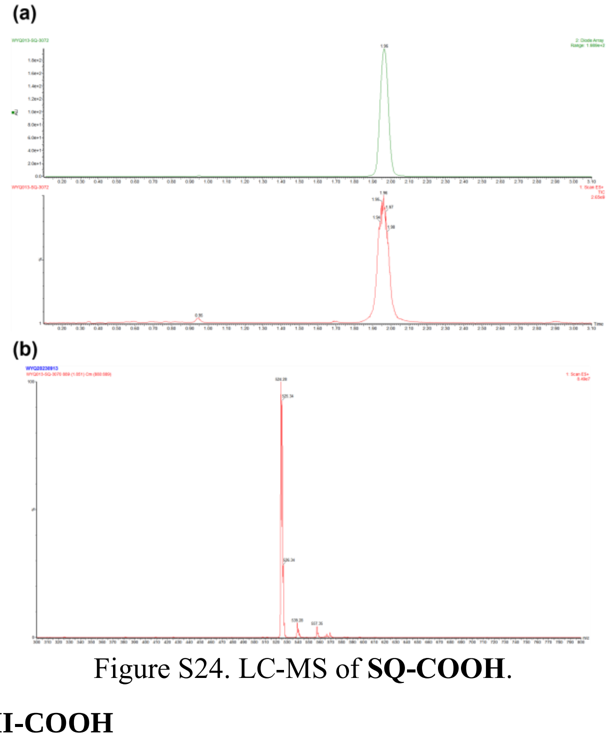

Figure S24. LC-MS of **SQ-COOH**.

c. Synthesis of NMI-COOH

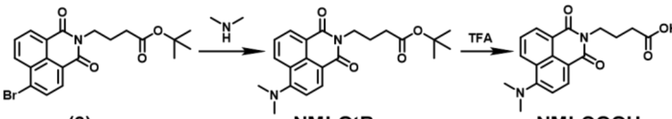

(3) NMI-OtBu NMI-COOH

(1) Synthesis of NMI-OtBu

Compound 3 was synthesized according to our previous paper.[3] **3** (100 mg, 0.239 mmol) was dissolved in 5 mL DMSO. 2 M dimethylamine THF solution (1.2 mL, 2.39 mmol) was then added. The reaction mixture was heated at 100°C for 12 h under N_2. After cooled down, 50 mL 1M HCl solution was added. The resulted solution was extracted by DCM (200 mL) and washed with 1M HCl (50 mL×2). The organic phase was dried over Na_2SO_4 and dried under vacuum to obtain **NMI-OtBu** as an orange solid (80 mg, 87%).

^{1}H NMR (400 MHz, $CDCl_3$) δ 8.56 (dd, 1H), 8.46 (m, 2H), 7.65 (dd, 1H), 7.15 (d, 1H), 4.19 (t, 2H), 3.11 (s, 6H), 2.32 (t, 2H), 2.00 (t, 2H), 1.40 (s, 9H).

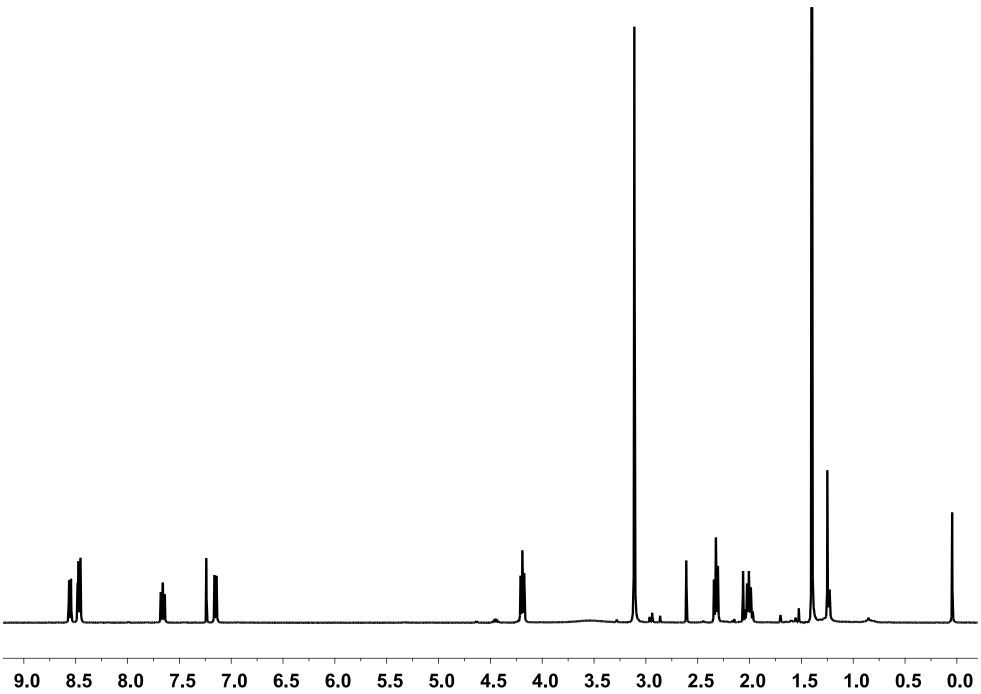

Figure S25. ^{1}H NMR (400 MHz, $CDCl_3$) spectrum of **NMI-OtBu**.

(2) Synthesis of NMI-COOH

NTI-OtBu (60 mg, 0.157 mmol) was dissolved in 2 mL DCM, and 0.5 mL TFA was added afterwards. The reaction was left at room temperature for 1 h. The solvent was evaporated under reduced pressure. Then the resulted solid was redissolved in acetone (1 mL) and precipitated in hexane (10 mL×2) to obtain **NMI-COOH** as an orange powder (50 mg, 95%).

^{1}H NMR (400 MHz, DMSO-d_6) δ 8.50 (dd, 1H), 8.45 (dd, 1H), 8.34 (d, 1H), 7.75 (dd, 1H), 7.21 (d, 1H), 4.06 (t, 2H), 3.09 (s, 6H), 2.28 (t, 2H), 1.87 (t, 2H).

$[M+H]^+$ calcd. for $C_{18}H_{19}N_2O_4^+$, m/z 327.13; found m/z 327.15.

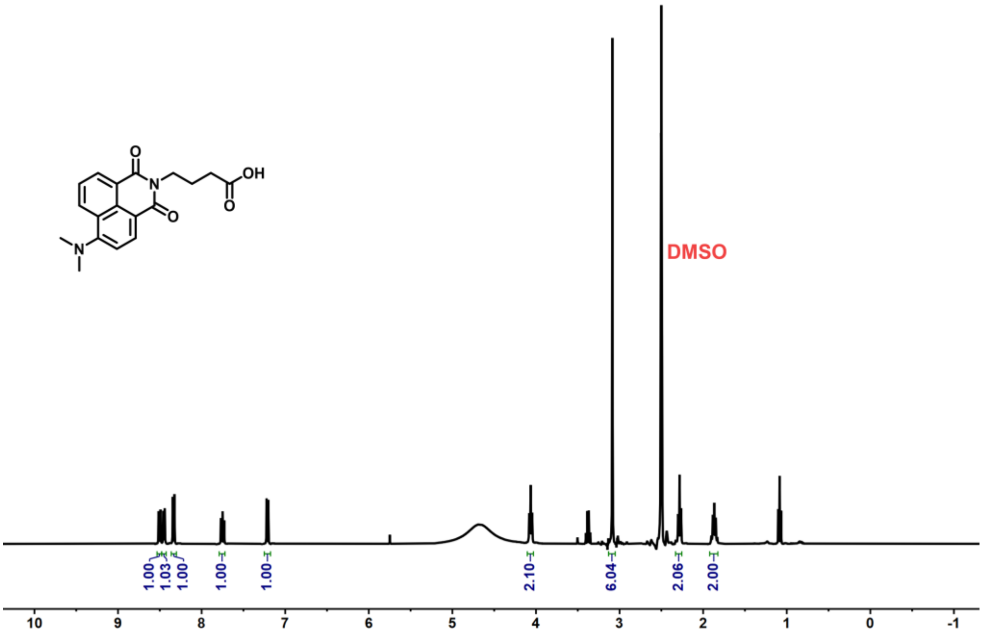

Figure S26. ^{1}H NMR (400 MHz, DMSO-d_6) spectrum of **NMI-COOH**.

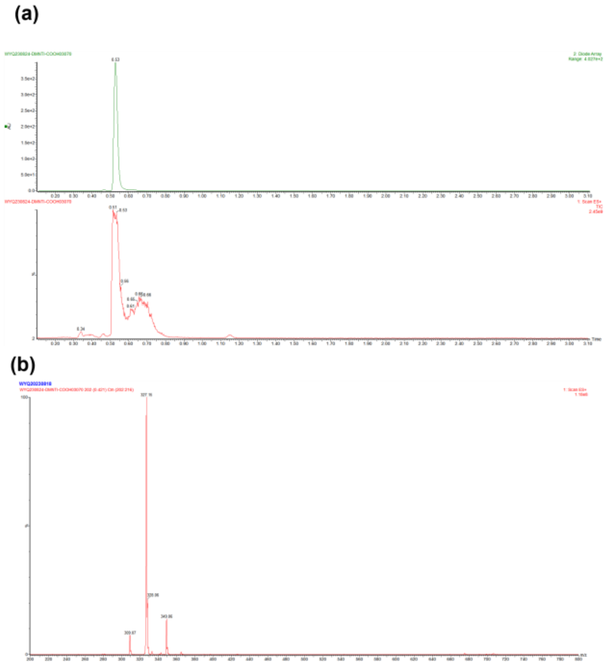

Figure S27. LC-MS of **NMI-COOH**.

d. Synthesis of CP-Dyes

CP-NH_2 (1 equiv.) and Dye-COOH (1.5 equiv.) were dissolved in DMF, followed by the addition of HATU (1.5 equiv.) and NMM (3 equiv.). The reaction was left for 24 h. The DMF

solution was then precipitated in a mixed solvent of diethyl ether/THF and washed twice to obtain **CP-Dyes**, which were characterized by LC-MS with high purity (Figure S26-S33). **CP-Cy3**, **CP-Cy5**, and **CP-Cy7** were obtained from our previous work[4].

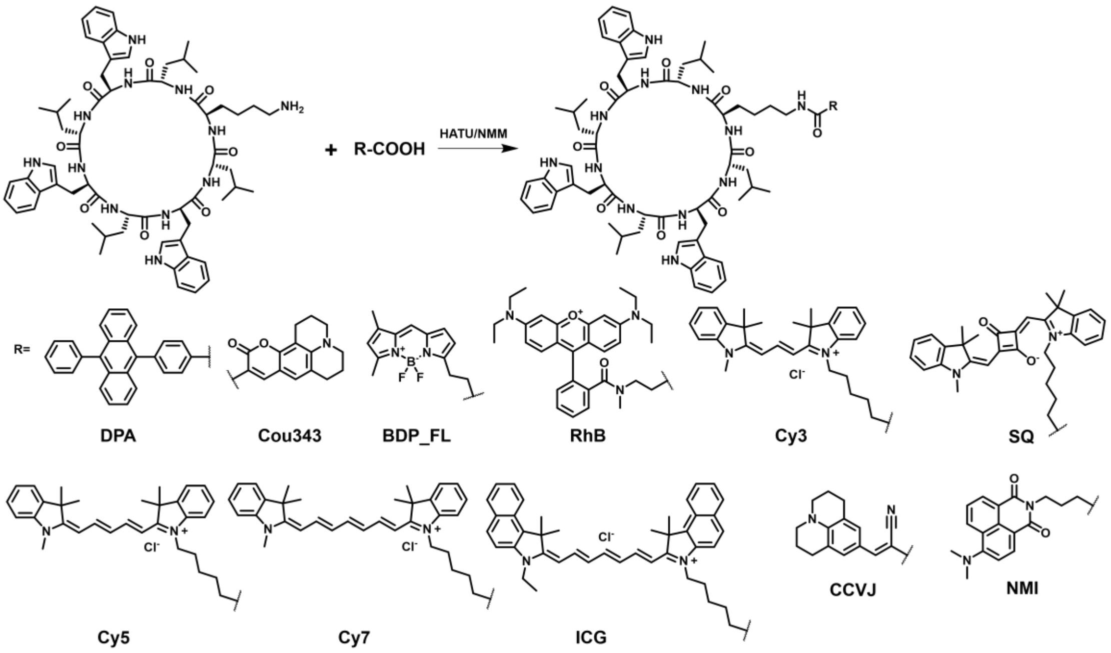

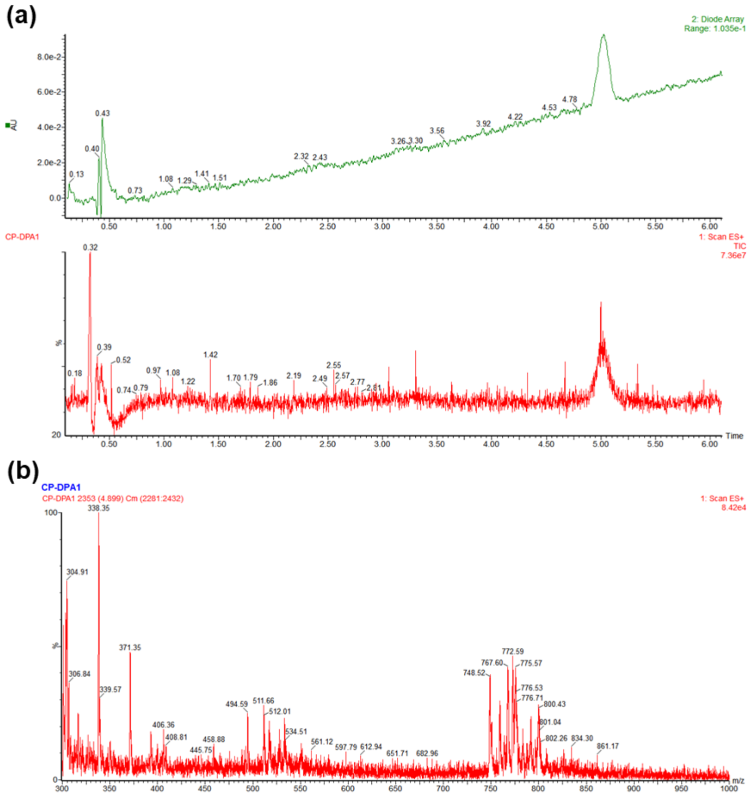

Figure S28 LC-MS analysis of **CP-DPA** (a) LC profile using UV detector (top) and MS detector (bottom); (b) mass spectrum of the LC peak (m/z=748.5, $[C_{90}H_{104}N_{12}O_9]^{2+}$: 748.4).

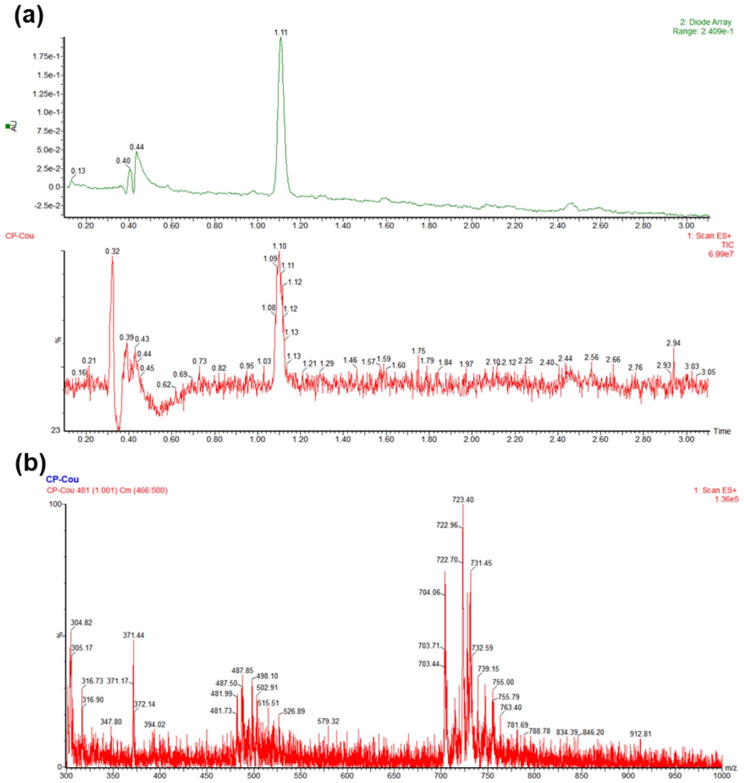

Figure S29 LC-MS analysis of **CP-Cou343** (a) LC profile using UV detector (top) and MS detector (bottom); (b) mass spectrum of the LC peak (m/z=703.4, $[C_{79}H_{101}N_{13}O_{11}]^{2+}$: 703.9).

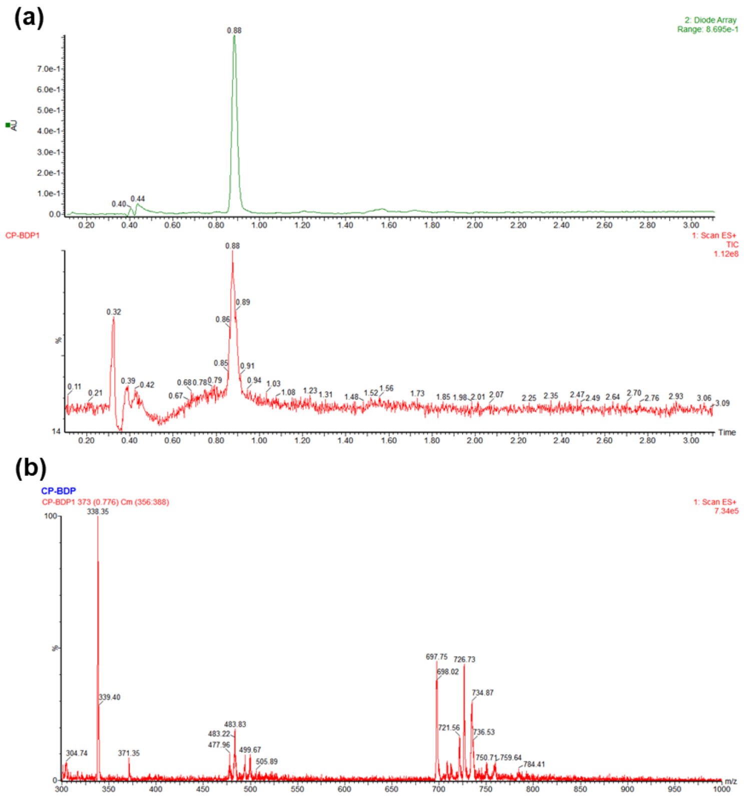

Figure S30 LC-MS analysis of **CP-BDP_FL** (a) LC profile using UV detector (top) and MS

detector (bottom); (b) mass spectrum of the LC peak (m/z=697.8, $[C_{77}H_{102}BFN_{14}O_9]^{2+}$: 698.4).

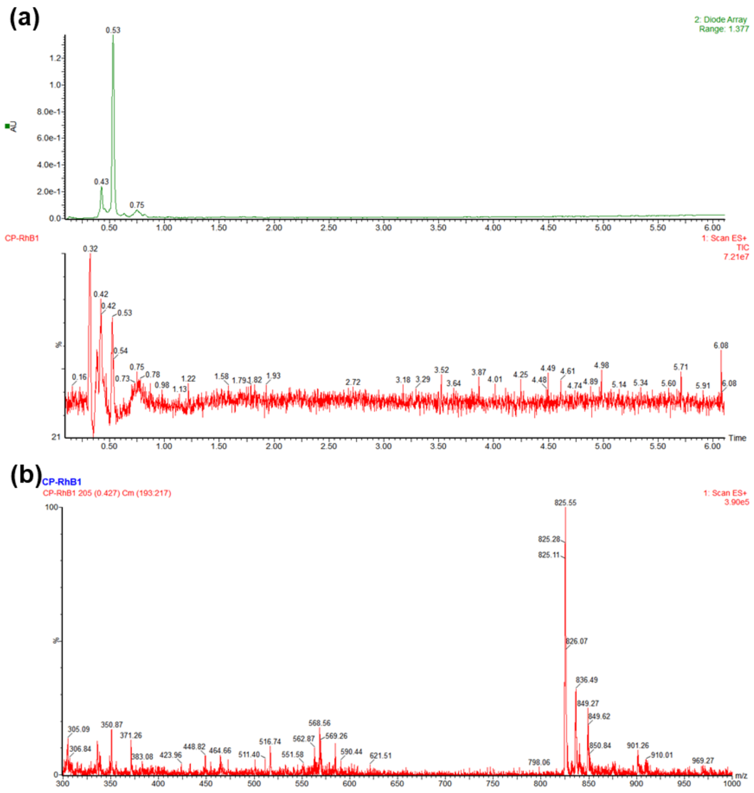

Figure S31 LC-MS analysis of **CP-RhB** (a) LC profile using UV detector (top) and MS detector (bottom); (b) mass spectrum of the LC peak (m/z=825.1, $[C_{95}H_{123}N_{15}O_{11}]^{2+}$: 825.5).

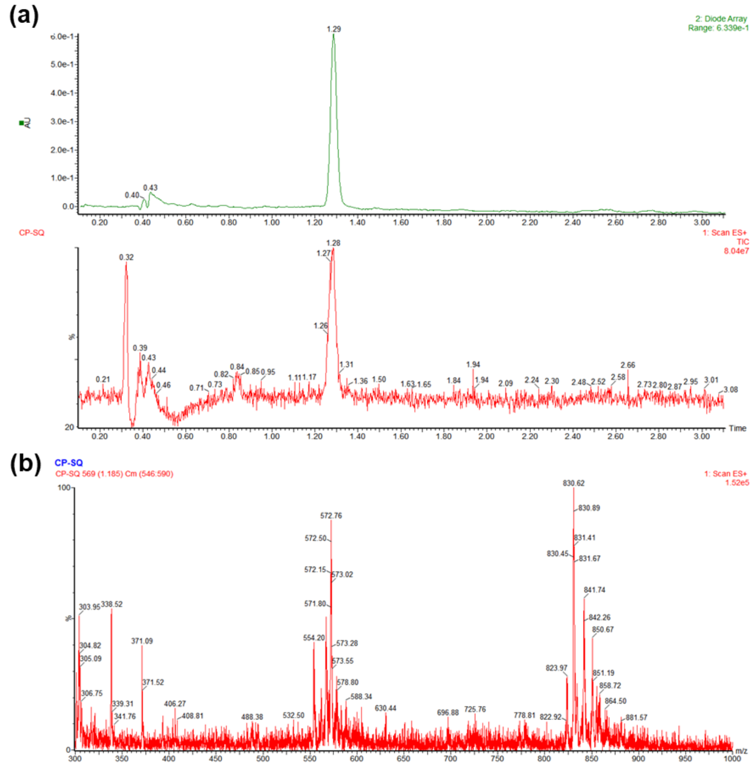

Figure S32 LC-MS analysis of **CP-SQ** (a) LC profile using UV detector (top) and MS

detector (bottom); (b) mass spectrum of the LC peak (m/z=824.0, $[C_{96}H_{122}N_{14}O_{11}]^{2+}$: 823.9).

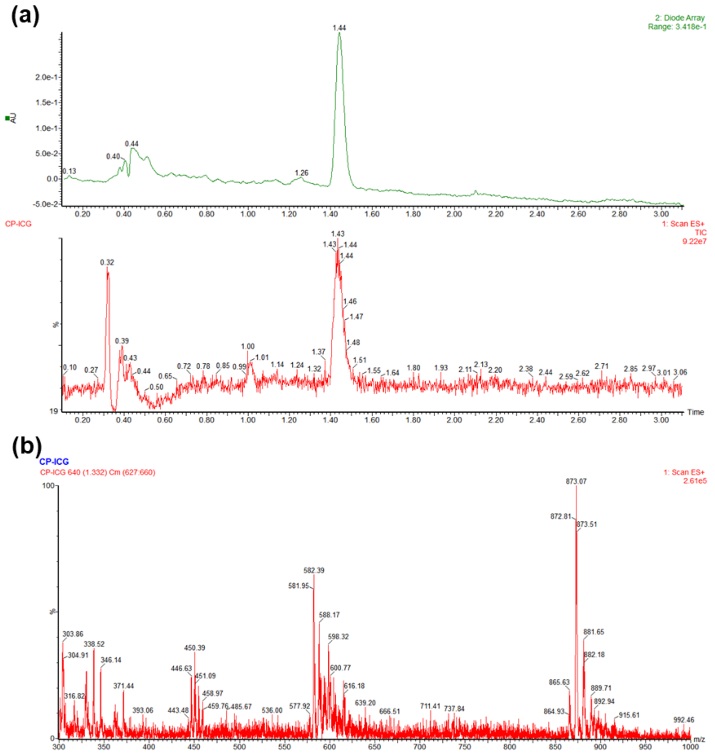

Figure S33 LC-MS analysis of **CP-ICG** (a) LC profile using UV detector (top) and MS detector (bottom); (b) mass spectrum of the LC peak (m/z=872.8, $[C_{106}H_{132}N_{14}O_{9}]^{2+}$: 873.0).

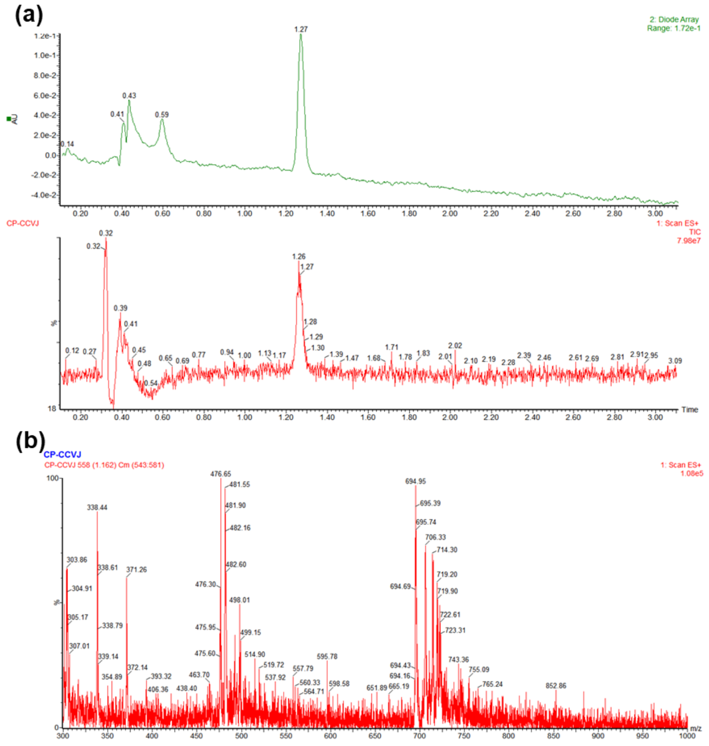

Figure S34 LC-MS analysis of **CP-CCVJ** (a) LC profile using UV detector (top) and

MS detector (bottom); (b) mass spectrum of the LC peak (m/z=694.7, $[C_{79}H_{102}N_{14}O_9]^{2+}$: 695.4).

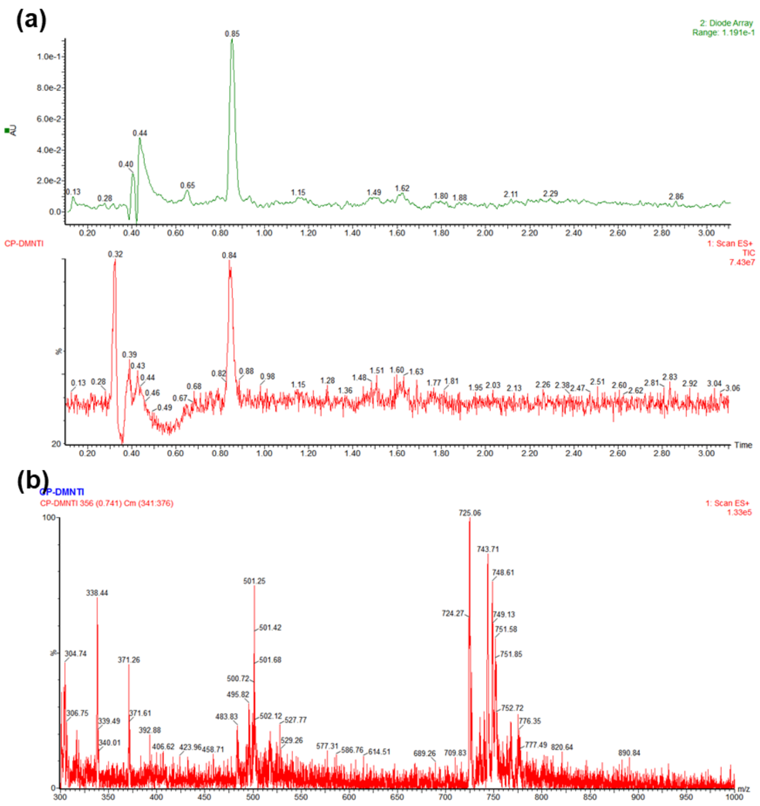

Figure S35 LC-MS analysis of **CP-NMI** (a) LC profile using UV detector (top) and MS detector (bottom); (b) mass spectrum of the LC peak (m/z=724.3, $[C_{81}H_{104}N_{14}O_{11}]^{2+}$: 724.4).

S9. References

[1] S. C. Larnaudie, J. C. Brendel, K. A. Jolliffe, S. Perrier, *J. Polym. Sci. Part A: Polym. Chem.* **2016**, *54*, 1003-1011.

[2] J. Karpenko, A. S. Klymchenko, S. Gioria, R. Kreder, I. Shulov, P. Villa, Y. Mély, M. Hibert, D. Bonnet, *Chem. Commun.* **2015**, *51*, 2960-2963.

[3] Q. Song, S. Goia, J. Yang, S. C. L. Hall, M. Staniforth, V. G. Stavros, S. Perrier, *J. Am. Chem. Soc.* **2021**, *143*, 382-389.

[4] H. Lu, Y. Wang, S. K. Hill, H. Jiang, Y. Ke, S. Huang, D. Zheng, S. Perrier, Q. Song, *Angew. Chem. Int. Ed.* **2023**, *62*, e202311224.
